# Bi-modal Distribution of the Second Messenger c-di-GMP Controls Cell Fate and Asymmetry during the *Caulobacter* Cell Cycle

**DOI:** 10.1371/journal.pgen.1003744

**Published:** 2013-09-05

**Authors:** Sören Abel, Tabitha Bucher, Micaël Nicollier, Isabelle Hug, Volkhard Kaever, Pia Abel zur Wiesch, Urs Jenal

**Affiliations:** 1 University of Basel, Biozentrum, Basel, Switzerland; 2 Hannover Medical School, Institute of Pharmacology, Hannover, Germany; 3 Brigham and Women's Hospital/Harvard Medical School, Global Health Equity, Boston, Massachusetts, United States of America; University of Geneva Medical School, Switzerland

## Abstract

Many bacteria mediate important life-style decisions by varying levels of the second messenger c-di-GMP. Behavioral transitions result from the coordination of complex cellular processes such as motility, surface adherence or the production of virulence factors and toxins. While the regulatory mechanisms responsible for these processes have been elucidated in some cases, the global pleiotropic effects of c-di-GMP are poorly understood, primarily because c-di-GMP networks are inherently complex in most bacteria. Moreover, the quantitative relationships between cellular c-di-GMP levels and c-di-GMP dependent phenotypes are largely unknown. Here, we dissect the c-di-GMP network of *Caulobacter crescentus* to establish a global and quantitative view of c-di-GMP dependent processes in this organism. A genetic approach that gradually reduced the number of diguanylate cyclases identified novel c-di-GMP dependent cellular processes and unraveled c-di-GMP as an essential component of *C. crescentu*s cell polarity and its bimodal life cycle. By varying cellular c-di-GMP concentrations, we determined dose response curves for individual c-di-GMP-dependent processes. Relating these values to c-di-GMP levels modeled for single cells progressing through the cell cycle sets a quantitative frame for the successive activation of c-di-GMP dependent processes during the *C. crescentus* life cycle. By reconstructing a simplified c-di-GMP network in a strain devoid of c-di-GMP we defined the minimal requirements for the oscillation of c-di-GMP levels during the *C. crescentus* cell cycle. Finally, we show that although all c-di-GMP dependent cellular processes were qualitatively restored by artificially adjusting c-di-GMP levels with a heterologous diguanylate cyclase, much higher levels of the second messenger are required under these conditions as compared to the contribution of homologous c-di-GMP metabolizing enzymes. These experiments suggest that a common c-di-GMP pool cannot fully explain spatiotemporal regulation by c-di-GMP in *C. crescentus* and that individual enzymes preferentially regulate specific phenotypes during the cell cycle.

## Author Summary

Bacterial processes like virulence, motility or biofilm formation are governed by the second messenger c-di-GMP. In most bacteria, c-di-GMP is produced and degraded by a complex network comprising dozens of enzymes. This has hindered a comprehensive analysis of the cellular role of c-di-GMP. Here we mutate the entire c-di-GMP network in *Caulobacter crescentus*, a model organism with inherent cell polarity and bimodal life-cycle. We find that a c-di-GMP free strain (cdG^0^) shows severe developmental defects, a loss of cell polarity and defective cell division. By determining c-di-GMP dose-response curves for individual processes and relating these to c-di-GMP levels, which were modeled for single cells progressing through the cell cycle, we define a quantitative frame for the c-di-GMP dependent program during the *C. crescentus* life cycle. We then show that the defects of cdG^0^ can be largely rescued by restoring c-di-GMP levels with a single heterologous enzyme producing c-di-GMP. However, much higher levels of the second messenger are required under these conditions as compared to the contribution of homologous enzymes. Our data argue for specific regulatory fine-tuning of the enzymes mediating c-di-GMP oscillation during the cell cycle and provide evidence for both global as well as insulated c-di-GMP pools.

## Introduction

Cyclic di-GMP is a ubiquitous second messenger that serves as key regulator of bacterial life-style decisions. While low intracellular concentrations of c-di-GMP promote a planktonic, single-cell life-style, where cells are generally motile and express virulence determinants, high levels of c-di-GMP lead to biofilm formation and persistence [1], [2]. Intracellular c-di-GMP levels are controlled by the antagonistic diguanylate cyclases (DGCs) and phosphodiesterases (PDEs) that either synthesize c-di-GMP from GTP or degrade it. These catalytic activities reside in GGDEF (DGC) and EAL or HD-GYP (PDE) domains, respectively. Typically, multiple proteins that contain GGDEF, EAL, and/or HD-GYP domains are encoded in the genome of a single bacterial species. In the most extreme cases, over 100 proteins are potentially involved in c-di-GMP metabolism, emphasizing the importance of c-di-GMP for bacterial signaling and adaptation [3]. This is also reflected by an ever-increasing number of established c-di-GMP receptors that regulate a wide range of cellular processes on the transcriptional, translational, or post-translational level [2], [4]. This includes the synthesis of virulence factors and toxins, the production of adhesins and biofilm matrix components, the regulation of different forms of cell motility, as well as cell cycle progression [2], [4]. Receptor affinities were reported from the low nM to the mid µM range (e.g. see [2], [5]–[11]). The physiological significance of such large differences in affinity is unclear.

In *Caulobacter crescentus*, the c-di-GMP mediated motile-sessile switch is integrated into a bimodal reproductive cycle, providing a simple and accessible cellular tool to study the c-di-GMP dynamics in time and space. *C. crescentus* divides asymmetrically to produce two daughters with distinct behavior and replication potential, a motile swarmer cell and a sessile stalked cell. The swarmer cell, equipped with a single polar flagellum and polar pili, remains in a motile but replication inert state for an extended period termed the G1-phase. The replication block is suspended concurrent with the transition of the swarmer cell into a stalked cell, during which the flagellar motor and the pili are lost and replaced by a stalk and an exopolysaccharide adhesin, the holdfast. At the same time, the density of the cells changes from a state of low to high buoyancy. Concurrently with these morphological changes, stalked cells proceed into S-phase to double their chromosomes and – after re-synthesizing a flagellum at the pole opposite the stalk - undergo an asymmetric cell division (G2-phase). Thus, *Caulobacter* cells continuously oscillate between different developmental and reproductive stages, offering an exemplary model system to dissect the molecular and cellular basis for the motile-sessile switch in bacteria and its coordination with cellular reproduction. This transition bears behavioral changes that are highly relevant for growth and persistence of many environmental and pathogenic bacteria. For example surface colonization and biofilm formation are key features of chronic infections of numerous human pathogens [12]. Just how exactly this behavioral change is staged and adjusted to the environment is not fully understood.

Several studies implicated that c-di-GMP is an important regulatory component of the *C. crescentus* developmental and cell cycle program [11], [13]–[17]. Different processes of pole development during the swarmer-to-stalked transition require c-di-GMP, including flagellar ejection, stalk elongation and holdfast biogenesis [13]–[17]. In addition, c-di-GMP interacts with the machinery that regulates the G1-S transition [11]. The *C. crescentus* genome encodes a total of 14 GGDEF/EAL domain proteins that are classified in three groups, GGDEF domain only, EAL domain only, and GGDEF-EAL composite proteins (Figure S1). The best-studied member of this group of proteins is PleD (CC2462), a DGC that is required for efficient pole remodeling during the motile-sessile transition. PleD is inactive in swarmer cells and is activated by phosphorylation during the swarmer-to-stalked cell differentiation [14], [18]. Intriguingly, activation of PleD is coupled to its subcellular sequestration to the differentiating pole, suggesting that PleD activates some nearby downstream effectors involved in pole remodeling [14], [19]. DgcB (CC1850) is an additional DGC involved in *C. crescentus* holdfast biogenesis and surface attachment during the swarmer-to-stalked cell transition. In contrast to PleD, DgcB is not controlled by cell cycle-dependent phosphorylation, but instead is antagonized in the swarmer cell by the PDE PdeA (CC3396). PdeA itself is only present in swarmer cells, where it counteracts DgcB and helps to keep the c-di-GMP levels low thereby licensing cell motility [15]. Specific proteolysis of PdeA during the cell cycle ‘releases’ DgcB activity to contribute to the sessile life style of the stalked cell [15]. DgcB also sequesters to the cell pole, again emphasizing a possible spatial coupling of controlled c-di-GMP production and the activation of downstream target processes [15]. Finally, DgcA (CC3285) was shown to possess DGC activity *in vitro* and *in vivo* but its physiological role is unknown [6]. At least two members of the GGDEF/EAL protein family, PopA (CC1842) and TipF (CC0710), are enzymatically inactive and have adopted novel roles [11], [16]. PopA is c-di-GMP specific effector proteins that binds c-di-GMP through its GGDEF domain and, in response, sequesters to the incipient stalked cell pole where it helps to recruit the replication initiation inhibitor CtrA to deliver it to the polar protease ClpXP [11]. The specific removal of CtrA then licenses cells for replication initiation (G1-S). The EAL domain protein TipF localizes to the pole opposite the stalk, where it contributes to the proper placement of the motor organelle in the polarized predivisional cell [16].

The proposed role of c-di-GMP in *C. crescentus* cell fate determination is consistent with the observed bimodal distribution of c-di-GMP during the cell cycle [18], [20]. Measurements of c-di-GMP indicated that motile swarmer cells and sessile stalked cells exhibit low and high levels of the signaling compound, respectively. Accordingly, the characteristic upshift of c-di-GMP during the SW-to-ST transition and the drop of c-di-GMP during birth of a new swarmer progeny are critical determinants of the differential developmental and replicative programs. However, this model raises several questions that need to be addressed. First, does c-di-GMP control additional cellular processes? Second, what are the molecular and cellular details of their execution in time and space? Third, which DGCs/PDEs are involved in the formation of c-di-GMP gradients during the *C. crescentus* cell cycle? Fourth, what is the minimal set of enzymes required to maintain c-di-GMP fluctuations, which in turn mediate oscillatory cell fate determination?

To address the above questions this study takes advantage of the moderate complexity of the c-di-GMP signaling network in *C. crescentus*. To generate a strain that is free of c-di-GMP, we have deleted all components that are potentially involved in the synthesis and degradation of the second messenger. We show that a c-di-GMP free mutant (cdG^0^) shows remarkable developmental and reproductive defects and looses morphological hallmarks of cell polarity. We then use this strain to re-construct the c-di-GMP signaling network, to functionally characterize the role of individual DGCs and PDEs and to generate a c-di-GMP dose response curve for individual c-di-GMP dependent processes using a heterologous *dgc* expression system. Our results indicate that different c-di-GMP dependent processes have distinct activation thresholds *in vivo* and provide strong evidence for a spatially structured mode of signaling.

## Results

**Figure 1:**
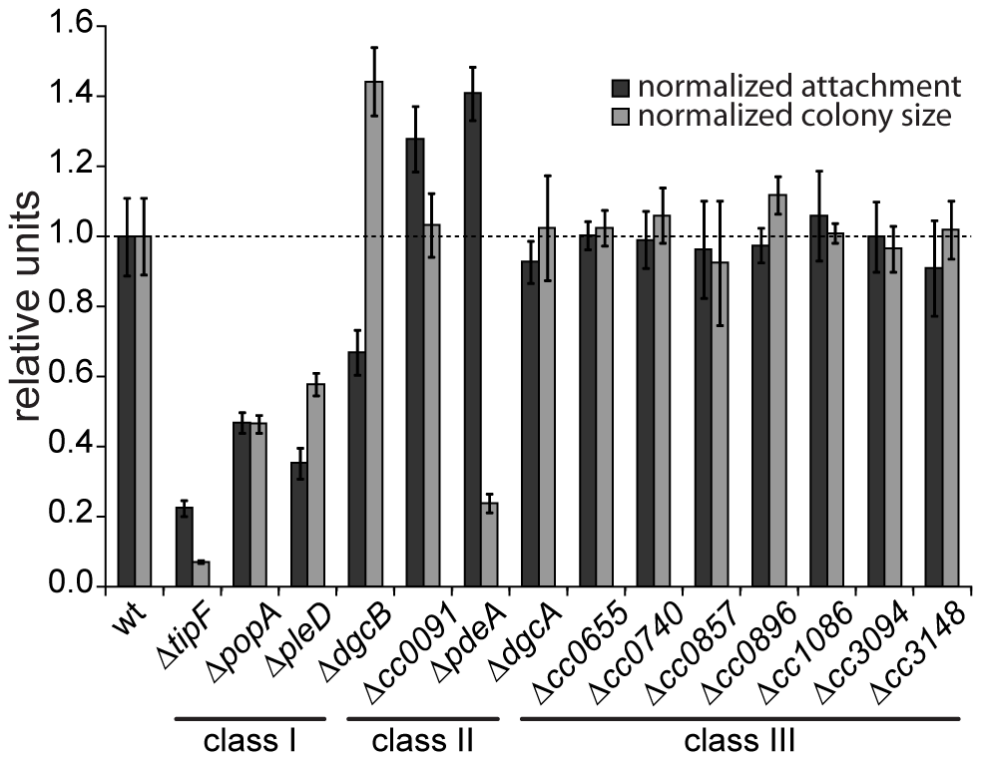
Motility and attachment behavior of *C. crescentus* is modulated by several GGDEF- and EAL-domain proteins. Surface attachment (black bars) and colony size on motility agar plates (grey bars) of mutants lacking individual GGDEF/EAL domain proteins are indicated relative to the wild type. Each bar represents the mean of seven independent experiments; the error bars represent the standard deviation; the dotted line indicates the wild-type behavior. The lines under the gene names outline the phenotypic classes. Class I: non-canonical behavior, class II: canonical behavior, class III: no phenotype (see main text for detailed information).

A systematic genetic analysis reveals the critical role of c-di-GMP for *C. crescentus* development. GGDEF/EAL domain proteins have been implicated in the antagonistic regulation of motility and attachment to surfaces in a wide variety of bacteria. To investigate the role of individual proteins with predicted diguanylate cyclase and/or phosphodiesterase activities in *C. crescentus*, individual deletions of all fourteen genes coding for GGDEF/EAL domain proteins (Figure S1) were generated. When scoring for motility and surface attachment, the two primary hallmarks of swarmer and stalked cell behavior, three classes of mutants were distinguished: First, strains with unaltered motility and attachment (8 out of 14); second, strains that show inverse alteration of motility and attachment, as expected for mutants lacking DGCs or PDEs, respectively (3); and third, strains exhibiting a non-canonical phenotype (3) (Figure 1). All eight proteins that, when absent, showed no apparent phenotype have conserved key residues required for enzymatic activity [21]. Thus, these proteins are not expressed or not active under the conditions tested, are redundant in function, or, alternatively, regulate more subtle cellular processes that cannot be easily scored with such general assays. Mutants with expected motile-sessile phenotypes include those with defects in DgcB and PdeA, two enzymes that were shown recently to direct cell fate in *C. crescentus* [15], and CC0091, a GGDEF-EAL domain protein that affects attachment but not motility. Three deletions, in *pleD*, *popA*, and *tipF*, reduce both motility and attachment. While PleD is a bona fide DGC required for the SW-to-ST cell transition [13], [14], PopA [11] and TipF ([16], unpublished data) are enzymatically inactive.

**Figure 2:**
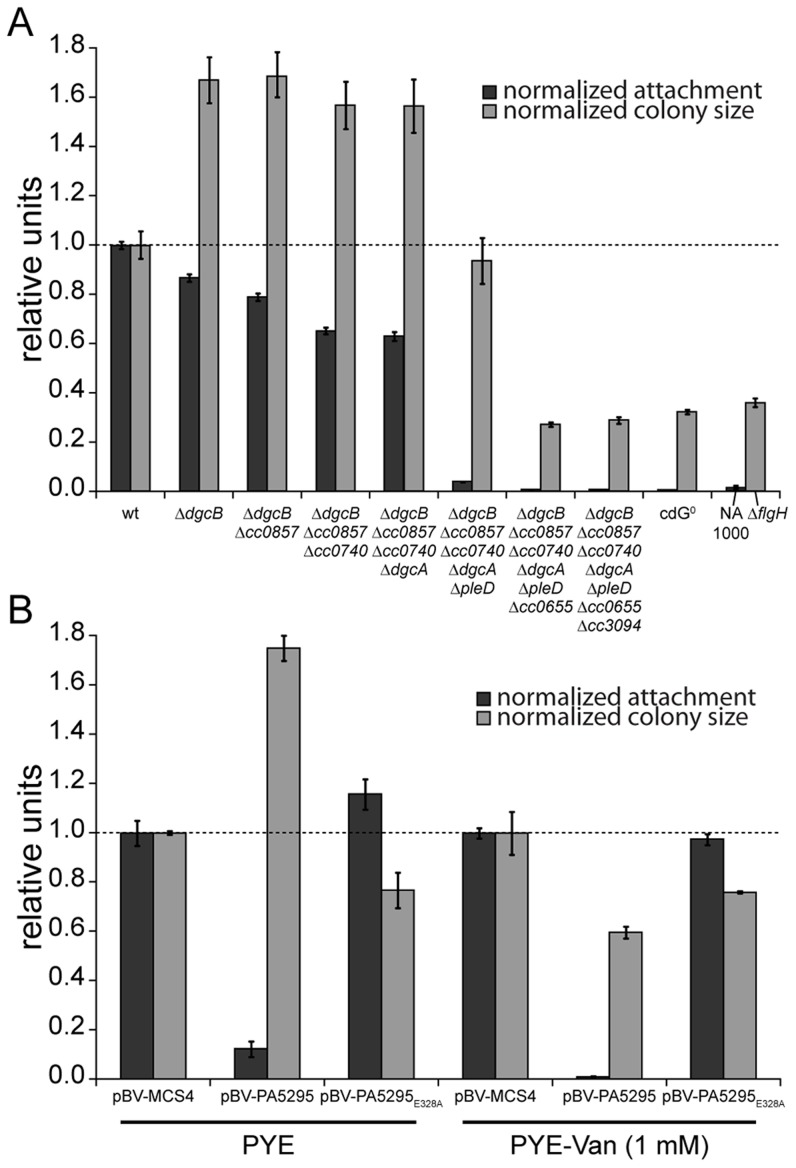
C-di-GMP is essential for motility and attachment in *C. crescentus*. A) A strain devoid of all potential diguanylate cyclases (cdG^0^ strain; CB15 Δ*cc0655* Δ*cc0740* Δ*cc0857* Δ*cc0896* Δ*cc3094* Δ*dgcA* Δ*dgcB* Δ*pleD*) was generated by cumulative deletions of genes that code for GGDEF domain proteins. Both surface attachment (black bars) and colony size on semi-solid agar plates as measure for motility (grey bars) of all mutant intermediates are shown normalized to the corresponding wild-type phenotype. Strain NA1000 and a Δ*flgH* mutant are shown as non-attaching and non-motile controls, respectively. The mean of eight experiments is given. B) Motility and attachment scores of wild-type *C. crescentus* strains carrying a plasmid expressing a heterologous phosphodiesterase (PA5295) under control of the inducible vanillate promoter. Each phenotype was normalized to cells carrying the empty plasmid backbone and compared to a strain expressing an active site mutant of the PDE, both under conditions with residual promoter activity (PYE) or full promoter activity (PYE-Van (1 mM)). The bars indicate the mean of six experiments; error bars represent the standard deviation; the dotted line indicates the wild-type behavior.

The observation that a notable amount of GGDEF and EAL domain proteins do not seem to be involved in the *C. crescentus* motile-sessile switch suggested an inherent redundancy in the c-di-GMP network. To uncover such a potential redundancy and to expose the entire range of c-di-GMP functionality during *C. crescentus* development, we decided to generate a mutant strain that lacks the second messenger altogether. To abolish the production of c-di-GMP, genes coding for GGDEF domain proteins were deleted consecutively. With the exception of those that encode known PDEs (PdeA, CC0091) or an enzymatically inactive protein (PopA) all GGDEF encoding genes were deleted. The deletions were done in two strain backgrounds, the CB15 wild type isolate [22] and NA1000, a lab adapted strain lacking holdfast [23]. Except for holdfast dependent phenotypes, the resulting strains showed identical behavior. The effect on surface attachment accumulated with increasing numbers of genes deleted, arguing that several DGCs cooperate to establish the sessile program (Figure 2A). Each of the early deletions resulted in a cumulative reduction of attachment with PleD having the strongest effect. In contrast, motility increased to a maximal level already after deletion of the first gene (*dgcB*), arguing that reducing c-di-GMP levels even lower cannot further boost flagellar motor function and, with regards to the results of the single gene deletions, that DgcB is the main DGC dedicated to motility regulation. Interestingly, in a strain accumulating more than five deletions, motility completely collapsed to a level of a non-flagellated mutant on motility agar plates (Figure 2A) and in liquid culture (data not shown), arguing that c-di-GMP regulates flagellar-based motility both positively and negatively. It is worth pointing out that while a single deletion of *CC0655* showed no effect on motility (Figure 1), this protein strongly contributes to cellular motility in a context where several other DGCs are absent. CC0655 might be required for a specific aspect of motility and, when inactivated, results in an overt phenotype only when other facets of motility are non-functional. Alternatively, DGCs could cumulatively contribute to the same aspect of motility. While the contribution of CC0655 is minor compared to other DGCs like PleD and DgcB, in a context where c-di-GMP is already low, its absence could “tip the balance” and reduce the c-di-GMP concentration below a threshold required to activate swimming of *C. crescentus*. In agreement with the prediction that a mutant lacking all eight DGCs is devoid of c-di-GMP, the second messenger was undetectable in extracts of this strain (Figure S2). In summary, a *C. crescentus* strain lacking c-di-GMP (cdG^0^) is completely non-motile and fails to attach to surfaces, arguing that c-di-GMP contributes to both the motile and the sessile cell program.

To confirm that these phenotypes depend on the activity of a DGC and not only on its presence, we expressed two active DGCs, PleD and DgcB, in the cdG^0^ strain as well as in single deletion mutants. Attachment and motility assays showed that both DGCs, but not their active site mutants could partially complement the defects of the cdG^0^ strain (Figure S3).

As an alternative approach to create a c-di-GMP free strain, we overexpressed the *Pseudomonas aeruginosa* PDE PA5295 in *C. crescentus* wild-type strain CB15 from a vanillate inducible promoter [11]. The observed phenotype resembles the phenotype of the cdG^0^ strain, in that both attachment and motility were reduced as compared to the wild type (Figure 2B). Surface attachment was completely abolished upon expression of PA5295, but not of its catalytically inactive mutant form PA5295_E328A_. In contrast, weak expression of PA5295 in the absence of the inducer vanillate increased motility, while in the presence of vanillate motility dropped below wild-type levels, but remained at a significantly higher level as compared to the cdG^0^ strain (Figure 2B). While this suggested that PA5295 is not able to completely deplete the c-di-GMP pool, these data strongly argue that the phenotype of the cdG^0^ strain is due to an overall drop in c-di-GMP concentration. Moreover, the data in Figure 2A and 2B implied that c-di-GMP directs surface attachment and motility control at different internal concentrations. While surface attachment and negative interference with motility require relatively high levels of c-di-GMP, low levels of c-di-GMP are sufficient to provide cells with the ability to swim.

### C-di-GMP is a key regulator of *C. crescentus* pole morphogenesis

**Figure 3:**
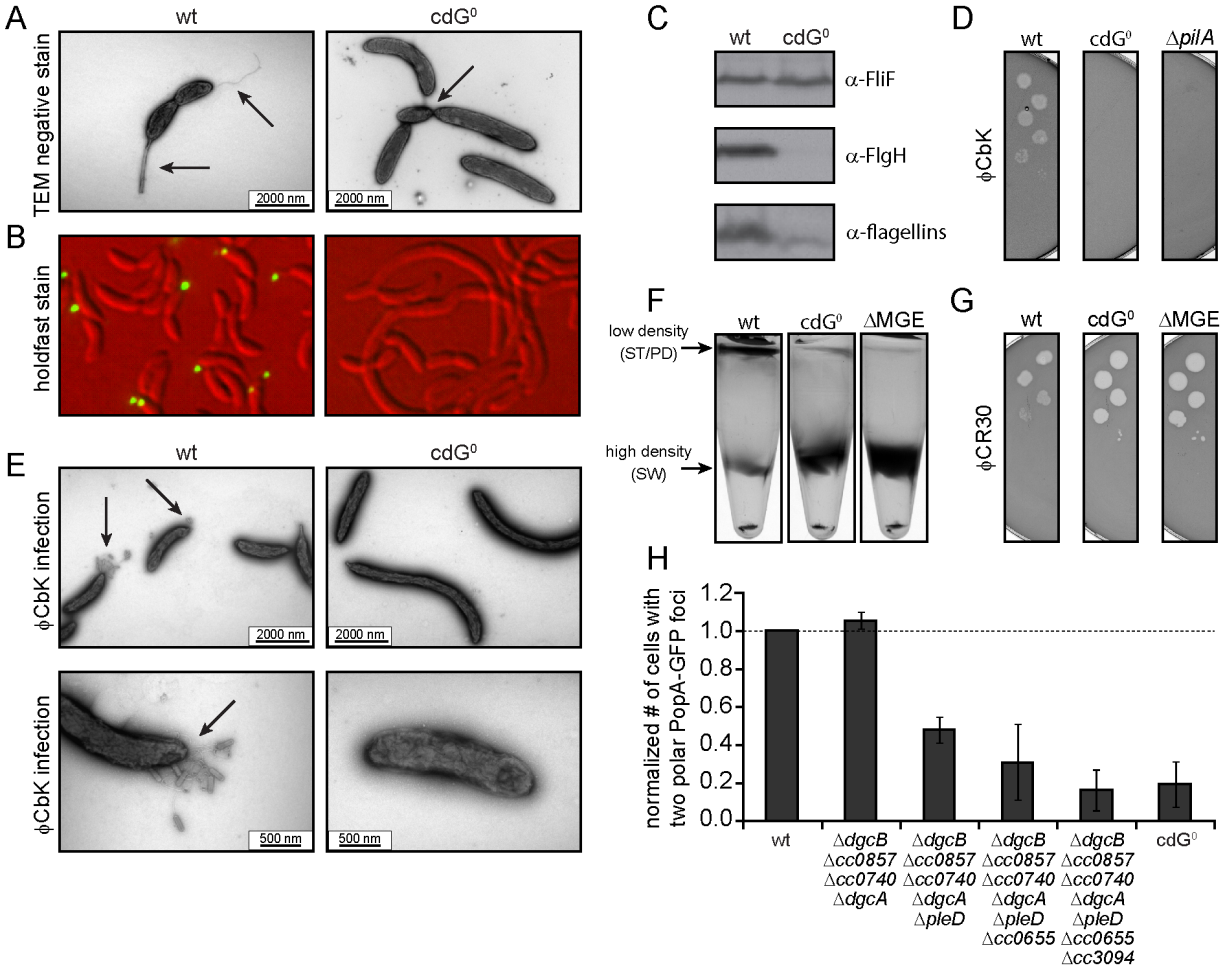
Depletion of c-di-GMP leads to severe deficiencies in development and cell morphology. A) Flagellum and stalk biogenesis: Representative transmission electron micrographs of wild-type (left panel) and cdG^0^ cells (right panel). Arrows highlight the flagellum, the stalk or a misplaced division septum, respectively. B) Holdfast biogenesis: Representative fluorescent micrographs of wild type (left panel) and cdG^0^ cells (right panel) after staining with fluorescently labeled wheat germ agglutinin. The holdfast specific lectin stain is shown in green and overlaid with a DIC image (red). C) Expression of late flagellar genes: The expression of representative flagellar proteins belonging to class II (FliF), class III (FlgH) and class IV (flagellins) of the flagellar hierarchy are analyzed in wild type (left) and the cdG^0^ strain (right) by immunoblots with specific antibodies. D) Pili-specific phage φCbK sensitivity: Plaque formation of a 1∶10 serial dilution of phage φCbK was assessed on a lawn of wild type (left), cdG^0^ strain (middle) and a *pilA* mutant (right) lacking the major pili subunit. E) Pili-specific phage φCbK sensitivity: Representative transmission electron micrographs of negatively stained wild type (left) and cdG^0^ strain (right) after brief exposure to the pili specific phage φCbK. Phage particles attached to the cell poles are highlighted by arrows. F) Cell type-specific cell density: Cells of the wild type (left), the cdG^0^ strain (middle) and a mutant lacking a mobile genetic element (MGE) [23] were separated by density gradient centrifugation. Arrows indicate the low- and high-density bands. The wild type low-density band contains a mixture of stalked (ST) and predivisonal (PD) cells while the high-density band consists of a homogenous population of swarmer (SW) cells. G) Protection from phage φCR30: Cell lawns of wild type (left), cdG^0^ strain (middle) and a mutant lacking a mobile genetic element (MGE) [23] were exposed to a 1∶10 serial dilution of φCR30. Please note that on *C. crescentus* wild type φCR30 forms turbid plaques, while the cdG^0^ or ΔMGE strains form clear plaques. H) PopA localization: The graph shows the quantification of fluorescent micrographs of cells expressing a PopA-GFP fusion. The bars represent the average number of cells that contain two polar foci. Data are given relative to the wild type. Error bars represent the standard deviation. At least 600 cells were quantified for each strain.

Next, we carried out a careful in-depth analysis of the cdG^0^ strain to define the role of c-di-GMP in *C. crescentus* development more precisely. Transmission electron microscopy revealed that the c-di-GMP free strain lacked flagella, offering an explanation for its non-motile phenotype (Figure 3A). In agreement with this, specific flagellar proteins are not synthesized in this background. Levels of proteins encoded by class III and class IV genes of the flagellar hierarchy [24] were either not present (FlgH) or strongly reduced (flagellins). In contrast, products of class II flagellar genes are present at normal concentrations (Figure 3C). Likewise, transcription of class II genes is largely unaltered in the cdG^0^ strain, while transcription of class III and class IV genes is severely reduced (Table 1).

**Table 1:** β-galactosidase activities of promoter *lacZ* fusions from flagellar genes.

Promoter activity in MillerUnits	wt	cdG^0^
P*_fliF_*-*lacZ*	980±188	1202±92
P*_flgH_*-*lacZ*	776±109	109±39
P*_fljL_*-*lacZ*	1387±112	36±25

Promoter activities are given in Miller units ± standard deviation. *fliF* = class II; *flgH* = class III; *fljL* = class IV flagellar gene.

Rapid and irreversible *C. crescentus* surface attachment depends on polar type IV pili and the presence of a polar adhesive holdfast exopolysaccharide [25], [26]. The latter can be visualized with fluorescent wheat germ agglutinin [27]. While *C. crescentus* wild type showed bright fluorescent staining at the stalked cell pole, no staining was observed for the cdG^0^ strain (Figures 3B). To detect the presence of pili, we employed the pilus specific phage φCbK [28]. Serial dilutions of φCbK form plaques on lawns of wild-type *C. crescentus*. In contrast, the sensitivity of the cdG^0^ strain is reduced to levels of a mutant lacking the major pilin subunit (Figure 3D). When *C. crescentus* cells were briefly exposed to the phage, fixed, and investigated with transmission electron microscopy, phage particles could readily be found at the pole of wild-type cells, where they decorated the polar pili. In the mutant lacking c-di-GMP, no phage particles were detected anywhere on the surface (Figure 3E). Together, these results indicate that the assembly of both adhesive organelles, pili and holdfast, requires c-di-GMP.

While analyzing the cdG^0^ strain we noticed that the different cell types could no longer be separated based on their different densities. Density gradient centrifugation allows separating low-density stalked and predivisional cells from high-density swarmer cells in the wild type. In contrast, all cells of the cdG^0^ mutant accumulated at the high-density position (Figure 3F), indicating that cell type specific density differences are dependent on the second messenger. Marks et al. [23] showed that differential cell density depends on a mobile genetic element (MGE) that is located on the chromosome and is linked to mucoidy and phage φCR30 susceptibility. A mutant lacking this mobile genetic element behaved like the cdG^0^ strain with respect to cell density (Figure 3F), phage sensitivity (Figure 3G), and mucoidy on sucrose containing agar plates (data not shown). Together this indicated that c-di-GMP regulates differential cell density in *C. crescentus* and that this process is linked to genes located on a mobile genetic element.

**Figure 4:**
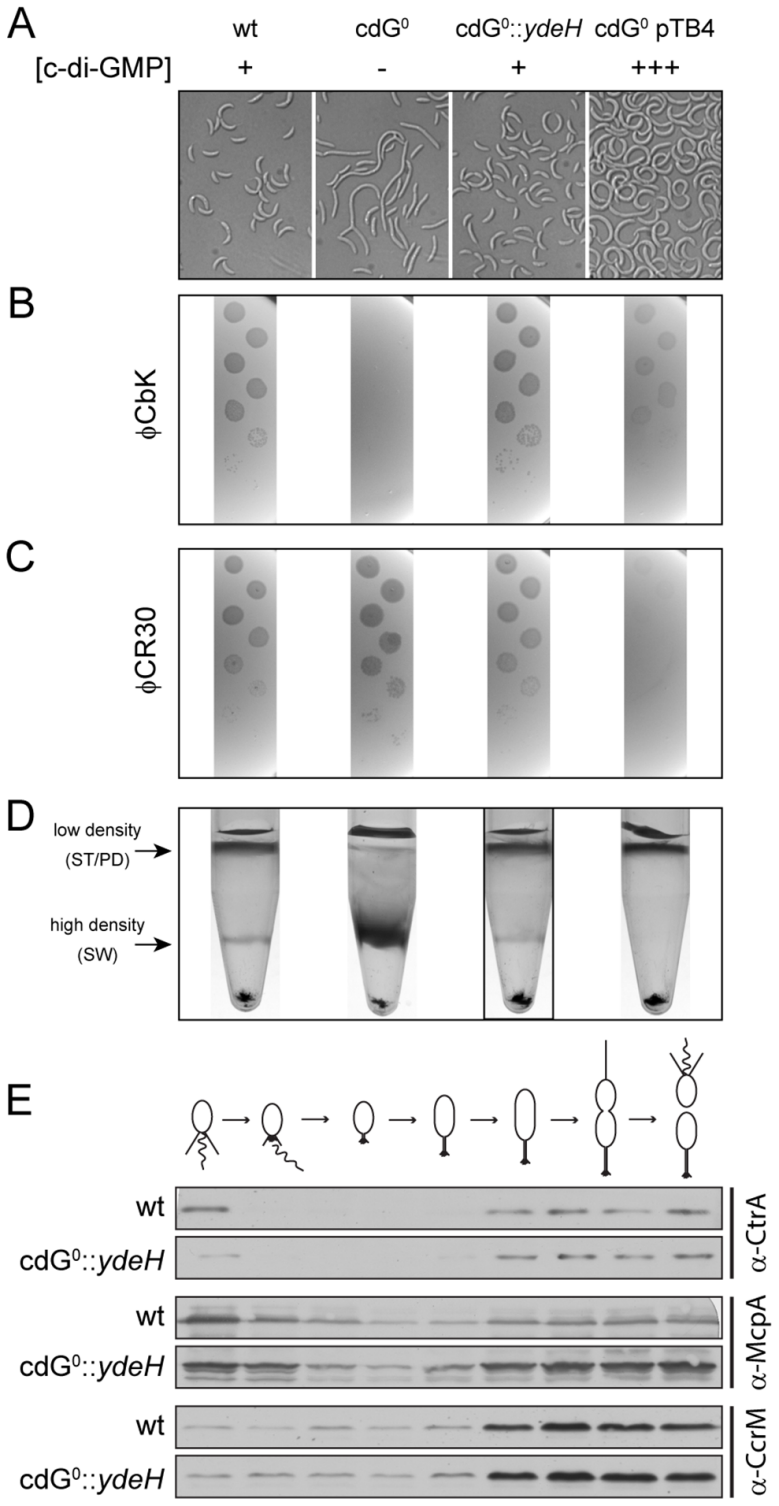
*In vivo* dose-response curves for c-di-GMP dependent processes. Cell morphology (A), phage sensitivity (B, C) and cell type-specific cell density (D) was recorded as a function of varying c-di-GMP concentration in a cdG^0^ strain expressing YdeH, a heterologous DGC. YdeH expression conditions and resulting c-di-GMP concentration are taken from Figure S2. See also Table 2 for an overview of the phenotypes with more c-d-GMP concentrations. A) *C. crescentus* cell length and morphology is controlled by c-di-GMP. Light micrographs of cells with increasing concentrations of c-di-GMP are shown. Wild-type cells carrying a control plasmid are shown for comparison. B–C) Interference with phage sensitivity at low and high c-di-GMP concentrations. Plaque assays are shown for lawns of cells with increasing concentrations of c-di-GMP with 1∶10 serial dilutions of the pili specific phage φCbk (B) and the S-layer specific phage φCR30 (C). D) Cell density is c-di-GMP dependent. *C. crescentus* cells with increasing intracellular c-di-GMP concentrations were separated by density gradient centrifugation. The resulting low- and high-density bands are highlighted. The black box marks the conditions (0.17 µM [c-di-GMP]) where swarmer cells from the high-density band were isolated for cell cycle synchrony analyses (Figure 4E). E) A heterologous DGC mediates normal cell cycle progression in *C. crescentus*. Cells derived from the cdG^0^::*ydeH* strain grown with intermediate levels of c-di-GMP (Figure 4D) were isolated from the high-density band, released into fresh medium containing IPTG and followed through a cell cycle. Immunoblots with specific antibodies directed against cell cycle regulated marker proteins were used to determine the homogeneity of isolated swarmer cells and their synchronicity during and progression through the cell cycle.

Microscopic images of the cdG^0^ strain also revealed characteristic morphology changes (Figures 3A, B, 4A). Cells lacked stalks and were often elongated with division septa frequently forming close to one end of the cell (Figure 3A). This suggested that c-di-GMP is important for morphological processes that are associated with proper re-direction of cell wall growth during the cell cycle.

We have recently exposed replication initiation as another c-di-GMP dependent cell cycle process. This process involves the GGDEF protein PopA (Figure S1), which, upon binding to c-di-GMP dynamically localizes to the old cell pole to deliver the replication initiation inhibitor CtrA to the polar protease ClpXP [11]. PopA also localizes to the new cell pole in a c-di-GMP independent manner [11]. As shown in Figure 3H, PopA localization to the stalked cell pole is unaffected in a mutant lacking the first four DGCs, but then gradually decreases with deletions of additional DGC genes. This emphasizes the importance of c-di-GMP for *C. crescentus* cell cycle progression and reiterates the redundant nature of DGCs for most of the c-di-GMP dependent processes.

Altogether, these data strongly imply that c-di-GMP is a critical regulatory determinant of *C. crescentus* cell polarity and cell fate determination, and that all processes involved in *C. crescentus* pole morphogenesis are regulated by the second messenger.

### Fine-tuning of cellular c-di-GMP levels by expression of a heterologous DGC

As indicated above, c-di-GMP is required for multiple developmental processes that need to be timed appropriately during the cell cycle. This raised the questions if these processes are mediated by cell cycle dependent changes of the c-di-GMP concentration, and how they respond to altered cellular levels of c-di-GMP. To address these questions, strains were constructed that allow the controlled expression of a heterologous DGC, YdeH from *E. coli*. For this, *ydeH* was expressed from the IPTG inducible *lac* promoter in single copy on the chromosome, on the low copy number plasmid pRK2 [29], or the medium copy number plasmid pBBR [30] (Figure S4). The combination of an inducible promoter and different copy numbers allowed fine-tuning of *ydeH* expression (Figure S5A) at constant levels during the *C. crescentus* cell cycle (Figure S5B). YdeH production was homogenous as expression differences at the single cell level were quite low and no sign for subcellular compartmentalization was detected (Figure S5C). Determination of the total c-di-GMP concentration [31] then allowed estimating the average intracellular c-di-GMP concentration at different levels of *ydeH* induction. For this, we determined the average cell volume (Figure S6A) from precise measurements of cell length (Figure S6B) and width (Figure S6C), as well as the relation between optical density and colony forming units (CFU) (Figure S6D). Using different *ydeH* expression constructs in the cdG^0^ strain and different inducer concentrations it was possible to vary the cellular c-di-GMP content from zero to approximately 60-fold of the average wild-type concentration, which was estimated to be about 130 nM (Figure S2).

To determine *in vivo* activation thresholds for specific c-di-GMP-dependent cellular processes, we next asked at which internal c-di-GMP levels individual processes are restored in the cdG^0^ strain. This includes cell morphology, φCbK and φCR30 phage sensitivity, cell density, motility and surface attachment. Interestingly, while most processes were restored to wild-type behavior at intermediate c-di-GMP levels, they showed distinct behavior at very low and very high c-di-GMP concentrations. This is illustrated for cell morphology in Figure 4A, S7A and Table 2. In the absence of c-di-GMP, cells are elongated, lack stalks and their characteristic curvature, and have misplaced division septa. At increasing second messenger concentrations, cells shorten and increase curvature until they are morphologically indistinguishable from the wild type. Upon further increase of the c-di-GMP concentration cells become even more curved and stalks and cell bodies continuously elongate. These morphological changes, and all other investigated phenotypes, are not influenced by IPTG, the inducer of *ydeH* expression (Figure S8A–F). Also, despite of this strong effect on cell morphology, cell growth was not affected by changing c-di-GMP levels (Figure S9).

**Table 2:** Assessment of cell curvature, phage sensitivity (φCbK and φCR30), and density switch with increasing c-di-GMP levels.

[c-di-GMP]/uM*	wt 0.13	cdG^0^ 0.00	0.08	0.17	0.21	0.25	0.35	0.53	0.64	2.78	5.07	7.79
**Cell curvature** **	+***	−−	−	+	+	+	+	+	+	++	+++	+++
**φCbK sensitivity**	+	−−−	+	+	+	+	+	+	+	−	−	−
**φCR30 sensitivity**	+	+++	+	+	+	+	+	+	+	−	−	−−
**amount of high density cells**	+	+++	+	+	+	+	+	+	−	−−	−−−	−−−

* YdeH expression conditions and resulting c-di-GMP concentrations are taken from Figure S2.

** See Figure 4 and S7 for primary data.

*** ‘+’ denotes wild-type like behavior; ‘++’ and ‘+++’ indicate a strong or very strong increase of the phenotype; ‘−’ and ‘−−’ indicate strong or very strong decrease of the phenotype; ‘−−−’ indicates the absence of plaque formation or high density cells, respectively.

A similar distribution was observed for the biogenesis of polar pili. While cells without c-di-GMP were completely resistant against the pili-specific phage φCbK, the lowest possible induction of YdeH restored phage sensitivity to wild-type levels (Figure 4B, S7B and Table 2). Changes in phage sensitivity occur at c-di-GMP concentrations where cell morphology is clearly still different from that of the wild type, arguing that the two processes differ with respect to c-di-GMP regulation. At high c-di-GMP levels phage sensitivity drops again with plaques becoming more turbid. Under these conditions, 10–100 times higher phage titers were required to form a visible plaque in the bacterial lawn. When challenging the cdG^0^ strain with phage φCR30 that uses the S-layer protein of *C. crescentus* as receptor, cells are hypersensitive to the phage. This is illustrated by clear and larger plaques and a 10-fold lower titer required for plaque formation as compared to the wild-type situation (Figure 4C). Similar to φCbK infections, small amounts of c-di-GMP restored normal phage sensitivity, while high c-di-GMP levels led to complete φCR30 resistance (Figure 4C, S7C and Table 2). Together, this suggested that c-di-GMP is critical for pili biogenesis but not for S-layer formation and that at high c-di-GMP levels another envelope structure conceals the surface exposed phage receptors. A possible candidate for such a structure is a capsule exopolysaccharide that could also be responsible for the cell type specific density difference of *C. crescentus* [23], [32]. Cell density behavior exactly parallels the φCR30 sensitivity pattern. In the absence of c-di-GMP, all cells accumulated in the high-density fraction, while the wild-type density distribution and synchronizability was restored at intermediate c-di-GMP levels, with high c-di-GMP levels forcing all cells into a low-density state (Figure 4D, S7D and Table 2). Expression of *ydeH* restored the density switch in a cell type specific manner as judged by the isolation of a pure population of swarmer cells, which when released into fresh media proceeded through the cell cycle synchronously, as indicated by the characteristic patterns of protein fluctuations (Figure 4E). It is worth emphasizing that cell cycle-dependent CtrA degradation, a process known to be regulated by c-di-GMP, is also fully functional in these cells (Figure 4E) [11], [15].

Together these findings indicate that c-di-GMP is required for the temporal and spatial regulation of developmental transitions and cell fate determination in *C. crescentus*, without affecting the overall growth rate under the conditions tested.

### Motility and surface attachment follow distinct c-di-GMP dose response curves

**Figure 5:**
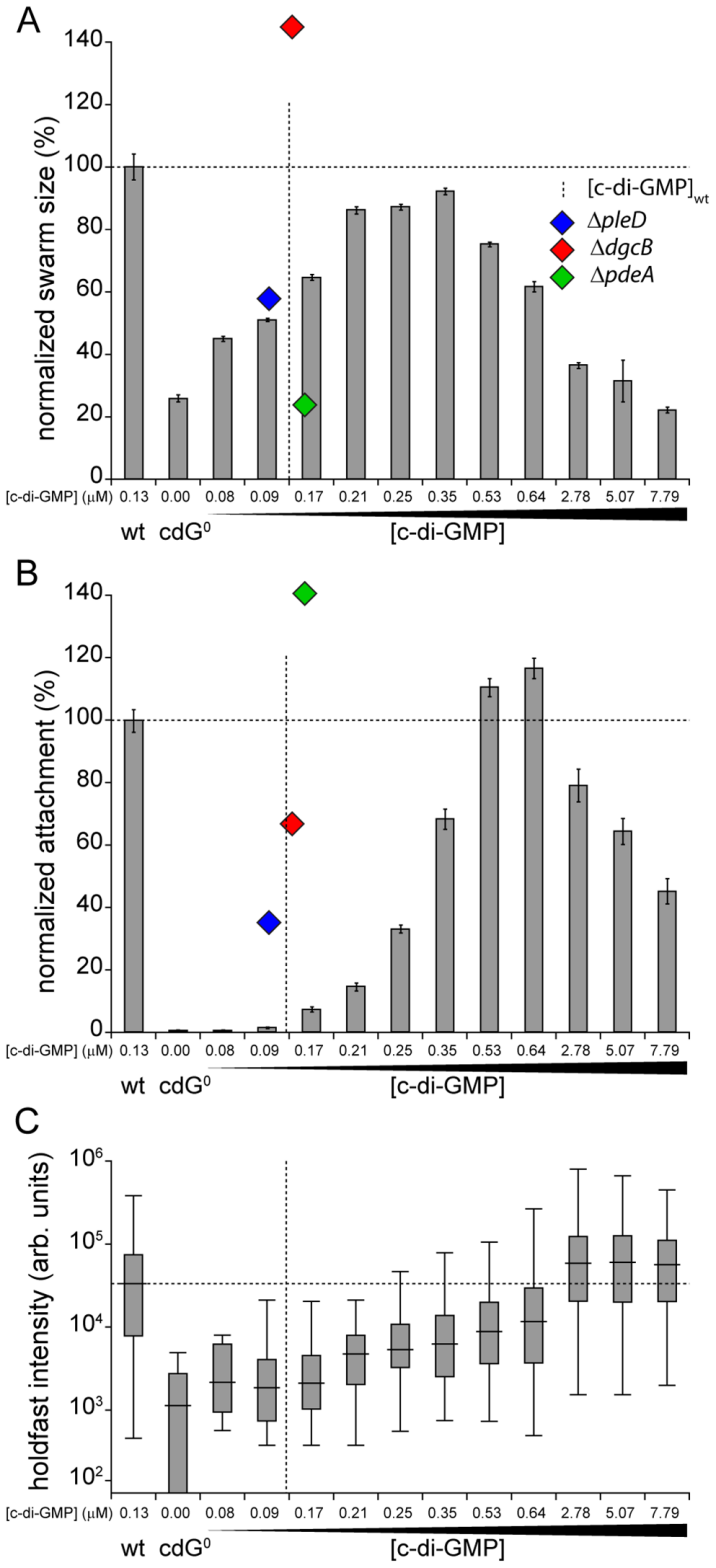
Motility and surface attachment show distinct *in vivo* c-di-GMP dose-response curves. Motility (A) and surface attachment (B) was recorded as a function of varying c-di-GMP concentration in a cdG^0^ strain expressing YdeH, a heterologous DGC. YdeH expression conditions and resulting c-di-GMP concentration are taken from Figure S2. The phenotypic behavior and c-di-GMP concentrations of mutants lacking selected DGCs or PDEs are indicated by blue (Δ*pleD*), red (Δ*dgcB*) and green diamonds (Δ*pdeA*). Holdfast production (C) was quantified as described in Materials and Methods with results represented as box plot. Big middle lines indicate the median holdfast fluorescence intensity of the sample. The box indicates the interquartile range and the whiskers include all data points not considered as outliers. The dotted lines highlight behavior and average c-di-GMP concentrations of *C. crescentus* wild type for comparison.

Because surface attachment and motility behavior can easily be quantified, we used these two c-di-GMP dependent processes to determine dose response curves. As shown in Figure 5, both curves follow an inverted U-shape, albeit with different peak positions. Without c-di-GMP cells fail to assemble a flagellum and hence are non-motile (Figure 5A). Flagellar biogenesis and motility was restored at relatively low intracellular c-di-GMP concentrations similar to the overall c-di-GMP levels in wild type. When c-di-GMP levels were increased further, motility again dropped until cells were completely non-motile on motility agar plates (Figure 5A) and also in liquid culture (data not shown). However, flagellar proteins are synthesized normally and flagellar biogenesis was not impaired (Figure S8G, H), arguing that motors are likely to be paralyzed under these conditions.

Similarly, the attachment defect of the cdG^0^ strain was restored with increasing *ydeH* expression strength. While motility was restored at relatively low levels of the second messenger, reconstituting surface attachment to wild-type levels required significantly higher c-di-GMP concentrations (Figure 5B). At low c-di-GMP concentrations surface attachment correlated well with the amount of holdfast produced under these conditions (Figure 5C) indicating that c-di-GMP dependent adhesin formation is a main factor driving surface colonization. As c-di-GMP levels reached their highest values, surface attachment dropped significantly, despite of increased holdfast production (Figure 5B, C). Several points about the data in Figure 5 are worth highlighting. First, flagellar biogenesis, motor interference and holdfast production are induced by c-di-GMP at distinct cellular concentrations, arguing for distinct *in vivo* activation thresholds of these processes. Second, despite of covering a wide range of c-di-GMP levels, the cdG^0^::*ydeH* strain failed to reach the same motility levels as observed for the wild type or for defined *C. crescentus* DGC mutants (e.g. Δ*dgcB*). Finally, when motility and attachment were scored in defined DGC and PDE mutants [15] large phenotypic changes were observed within a relatively narrow concentration window of the second messenger (Figure 5A, B). The observation that in the cdG^0^::*ydeH* strain the respective regulatory transitions occur in a much wider c-di-GMP concentration range argues that homologous components involved in cell cycle dependent c-di-GMP metabolism must be subject to specific regulatory fine-tuning that cannot be mimicked by the constitutive expression of a heterologous DGC.

### High intracellular c-di-GMP concentrations interfere with different cellular processes through multiple pathways

**Figure 6:**
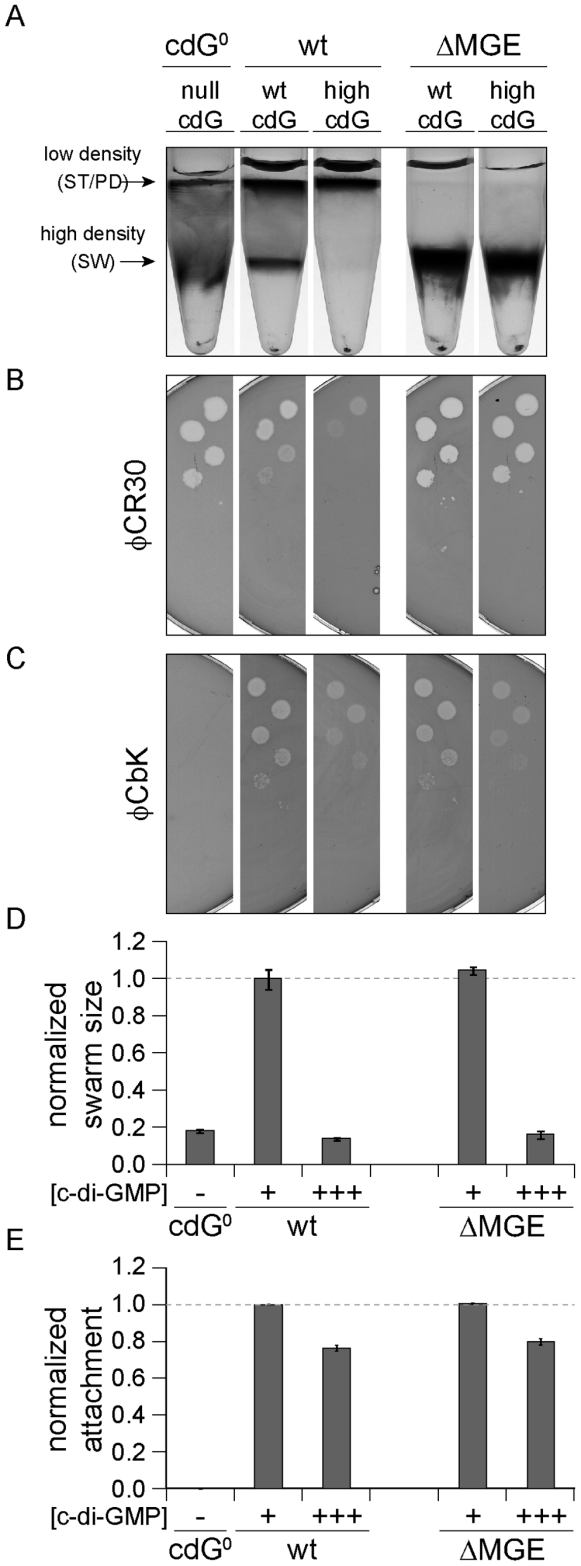
Cell density and φCR30 phage sensitivity are regulated by c-di-GMP via a mobile genetic element. YdeH was overexpressed ([c-di-GMP] +++) in *C. crescentus* wild type (wt) or in a mutant lacking the mobile genetic element (ΔMGE) [23] and strains were compared to isogenic strains lacking YdeH ([c-di-GMP] +) and to the cdG^0^ strain ([c-di-GMP] −). Differential cell density (A), sensitivity to phages φCR30 (S-layer) (B) and φCbK (pili) (C), colony size on motility plates (D), and surface attachment (E) were scored for all strains. The positions of high- and low-density bands after density gradient centrifugation are marked by arrows. The bars in the motility and attachment assays represent the mean of five or eight experiments, respectively. The error bars indicate the standard deviation. The quantified data were normalized to wild type without YdeH overexpression and the dotted lines indicate wild-type behavior. This figure is complemented by Figure S10 which includes more controls.

At very high c-di-GMP concentrations, we observed interference with all processes that we investigated. The observation that reduced surface attachment, phage sensitivity and differential cell density were all triggered at concentrations above 1 µM c-di-GMP raised the question if these changes result from the same underlying c-di-GMP-dependent process. φCR30 sensitivity and differential cell density were recently linked to a mobile genetic element (MGE) that contains candidate genes involved in the biosynthesis of capsule exopolysaccharides [23], [32]. If genes located on the mobile genetic element mediate the observed changes at high c-di-GMP levels, one would expect these phenotypes to become c-di-GMP insensitive in a mutant lacking the MGE. To test this, *ydeH* was overexpressed in *C. crescentus* wild type or in a strain carrying a deletion of the MGE region. Cells lacking the MGE failed to switch to the low-density state and were hypersensitive to phage φCR30 (Figures 6A, B, S10A, B) [23]. In the wild type, high c-di-GMP concentrations led to migration of all cells to the low-density band in a density-gradient centrifugation. In contrast, the same high c-di-GMP levels did not affect the phenotype of cells lacking the MGE. Similarly, high c-di-GMP concentrations increased the resistance against phage φCR30, while a strain lacking the MGE remained hypersensitive. In contrast, the presence of the mobile genetic element has no influence on c-di-GMP mediated φCbK sensitivity, motility and attachment (Figure 6C–E, S10C–E).

Together, this indicated that c-di-GMP affects components encoded by the MGE to modify cell density and φCR30 sensitivity. Furthermore, this pathway seems to be distinct from the regulatory mechanisms that govern a reduction of φCbK sensitivity, motility and attachment at high c-di-GMP levels.

### C-di-GMP levels oscillate during the cell cycle

**Figure 7:**
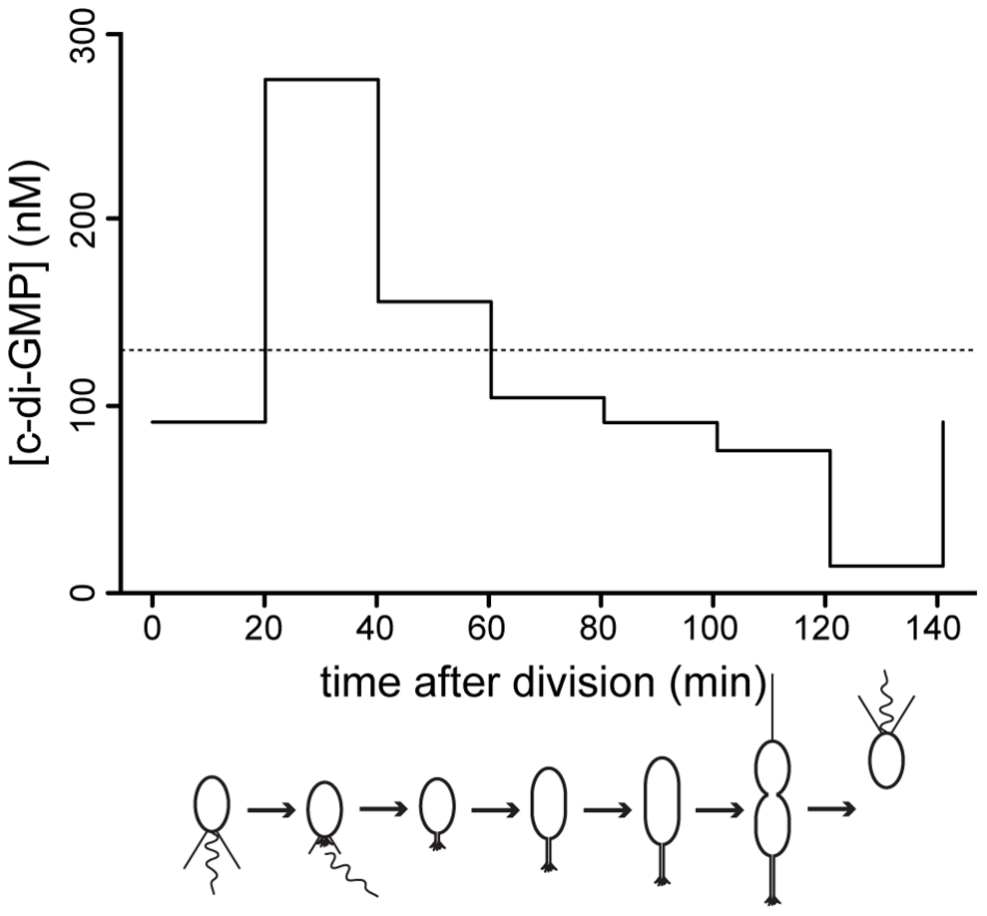
C-di-GMP oscillation during the *C. crescentus* cell cycle. The graph shows modeled c-di-GMP fluctuations in a single *C. crescentus* cell during a full cell cycle. The predictions are based on c-di-GMP measurements in synchronized populations of *C. crescentus* wild-type cells and on a mathematical model accounting for differences in cell age and cell cycle length of synchronized populations (Figure S11). The c-di-GMP concentration is given in nM and the progression of the cell cycle is given in minutes after division of the predivisional cell. Only the c-di-GMP concentration of the swarmer progeny is shown. Cell cycle progression is depicted schematically below the graph. The dotted line indicates the average c-di-GMP concentration measured in non-synchronized wild-type populations.

If c-di-GMP is homogenously distributed throughout the cytoplasm, changes in global c-di-GMP content should directly mediate changes in bacterial behavior. But how does the intracellular c-di-GMP concentration during the cell cycle compare to the measured dose-response curves? We determined the c-di-GMP concentration throughout the cell cycle using synchronized populations of cells (Figure S11A). Because our synchronization technique harvests all high-density swarmer cells irrespective of their exact age after division and because the cell cycle length of individual cells varies, experimentally determined c-di-GMP concentrations represent population averages rather than exact values corresponding to a distinct cell cycle stage. Knowing the population composition at each cell cycle time point (Figure S11B) would allow inferring the exact single cell concentration of c-di-GMP at any given time of the cell cycle. To obtain the population composition, we developed a mathematical model that describes the growth of a *C. crescentus* population and the relative age of its constituents (Figure S11C, D). This model was parameterized with measurements of cell cycle length variation and relative swarmer and stalked cell cycle lengths combined with growth curves during the respective experiment (see Materials and Methods). Numerical simulations yielded the population composition (Figure S11B), from which we calculated the internal c-di-GMP content (Figure 7). We find that the c-di-GMP concentration peaks during the swarmer-to-stalked cell transition, falls slowly to a lower level in the stalked cell and is lowest in swarmer cells just after division. Qualitatively, this pattern is in line with the observed phenotypes in the cdG^0^::*ydeH* strain, where holdfast formation occurs at very high levels, while processes taking place in the predivisional cell (e.g. pili and flagellum assembly) require intermediate levels, and motility being promoted by low c-di-GMP levels. However, c-di-GMP concentrations related to specific phenotypes are much lower in synchronized wild type cells as compared to the dose response curves in the cdG^0^::*ydeH* strain (Figure 5). For example even peak concentrations measured during the swarmer-to-stalked transition are too low to explain the behavior of populations of the cdG^0^::*ydeH* strain. This demonstrates that although a heterologous DGC can qualitatively restore all c-di-GMP dependent processes in a mutant strain lacking all homologous enzyme systems, significantly higher cellular c-di-GMP concentrations are needed in such a context. This again argues for a specific regulatory arrangement of c-di-GMP signaling components that permits the proper fine-tuning of processes driving differentiation and growth in *C. crescentus*.

### Redundant control of c-di-GMP oscillations during the cell cycle

**Figure 8:**
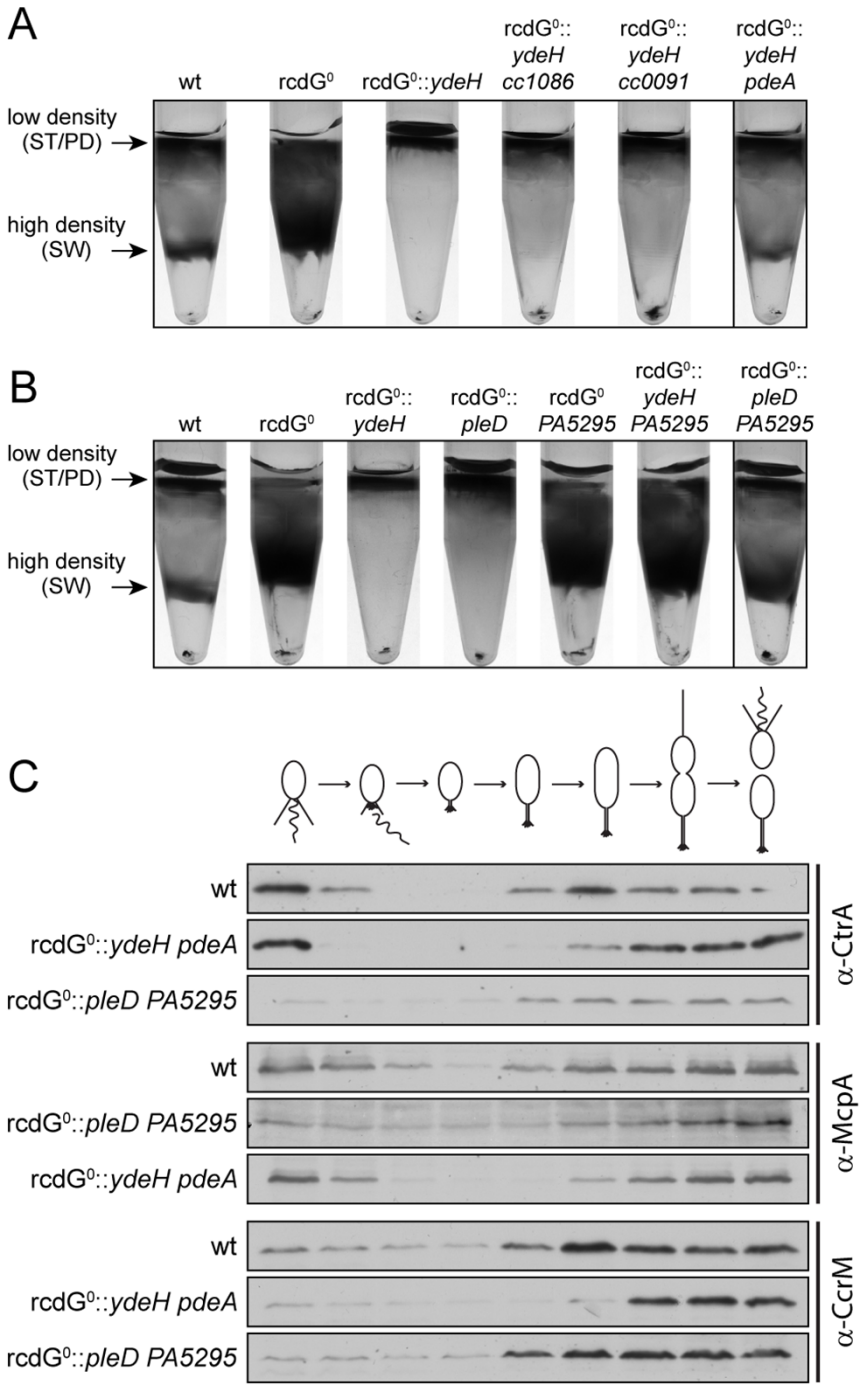
Redundant enzymes facilitate c-di-GMP fluctuations during the cell cycle. A) PdeA is sufficient to establish cell type-specific cell density distribution in the presence of a continuous source of c-di-GMP. *C. crescentus* cell density was analyzed by density gradient centrifugation for wild type cells (wt), a strain that lacks all endogenous diguanylate cyclases and phosphodiesterases (rcdG^0^), rcdG^0^ expressing the heterologous DGC YdeH, and rcdG^0^::*ydeH* complemented with three genes encoding homologous PDEs (CC1086, CC0091, PdeA). Cells were grown in the presence of 555 µM IPTG for YdeH induction. The position of the low- and high-density bands are marked with arrows and labeled with the cell types according to the fractionation behavior of wild type. The black box indicates the strain that was used to isolate swarmer cells for the analysis in Figure 8C. B) PleD is sufficient to establish cell type-specific cell density distribution in the presence of a constitutive PDE. Labels are like in (A). Note that although PA5295 was driven by the vanillate promoter its expression was not induced. Residual expression levels of the PDE were sufficient to destabilize c-di-GMP in this experiment. The black box indicates the strain that was used to isolate swarmer cells for the analysis in Figure 8C. C) Reconstitution of c-di-GMP fluctuations is sufficient for cell fate determination. Cells of the wild type (wt) and the diguanylate cyclase/phosphodiesterase free strain either expressing YdeH and PdeA (rcdG^0^::*ydeH pdeA*) or expressing PleD and PA5295 (rcdG^0^::*pleD* PA5295) were isolated from the high-density fraction of the gradient (see above) and released in fresh medium containing IPTG or vanillate, respectively. Samples were analyzed at 20 min intervals and probed with antibodies against cell cycle marker proteins (CtrA, McpA, and CcrM). Cell cycle progression is indicated schematically above the immunoblots.

To determine the minimal set of components required for c-di-GMP cell cycle fluctuations we made use of the observed c-di-GMP dependent density switch at the swarmer-to-stalked cell transition. While in the cdG^0^ strain all cells accumulate at the high density band, intermediate level expression of *ydeH* restored differential cell density, synchronizability and normal cell cycle progression in this background (Figure 4D, E). This strongly argues that c-di-GMP oscillation is at least partially restored under these conditions. Since YdeH is constitutively expressed and is unlikely subject to cell cycle regulation, the production of c-di-GMP in this strain should be constant. Normal cell cycle oscillation of this strain could thus be explained by varying sensitivities of downstream effectors during the cell cycle or by cell cycle-dependent breakdown of c-di-GMP by PDEs. To distinguish between these possibilities, we deleted all genes encoding potential PDEs (*cc1086*, *cc0091*, *CC3148* and *pdeA*) in the cdG^0^ strain, thereby generating a strain lacking all enzymes involved in c-di-GMP metabolism (rcdG^0^). This strain was phenotypically indistinguishable from the cdG^0^ strain (data not shown). In particular, all cells accumulated in the high-density fraction during density gradient centrifugation. However, when introducing a single copy of *ydeH* into the chromosome of this strain, we observed that low levels of YdeH expression already lead to a complete shift of cells to the low-density fraction (Figure 8A). This excluded the possibility of varying effector sensitivities mediating cell type specific density and indicated that one or several PDEs are responsible for cell-cycle dependent c-di-GMP fluctuations in the cdG^0^::*ydeH* strain.

When *cc1086*, *cc0091* or *pdeA* were re-introduced into their original chromosomal loci of the rcdG^0^::*ydeH* strain, only *pdeA* was able to restore differential cell density (Figure 8A). Light microscopy analysis confirmed that the high-density band of this strain contains a pure population of swarmer cells (data not shown). Moreover, when these cells were followed over time, the characteristic fluctuations of several indicator proteins confirmed their synchronous progression through the cell cycle (Figure 8C). We have recently shown that PdeA is a swarmer cell-specific PDE [15]. This argues that a constant source of c-di-GMP (originating from YdeH) and a swarmer cell specific PDE is sufficient to establish c-di-GMP oscillations leading to proper cell type-specific cell density. We next asked if a DGC, which is subject to cell cycle regulation, was able to create the same fluctuations and restore cell cycle timing. For this, we re-introduced the *pleD* gene into the rcdG^0^ strain. PleD is a cell-cycle controlled DGC that is inactive in swarmer cells [14], [19]. Similar to the constitutive YdeH, PleD derived c-di-GMP led to an accumulation of low-density cells in this strain background (Figure 8B). Limiting the production of c-di-GMP to specific times during the cell cycle alone is therefore not sufficient to establish the cell type specific program. However, when we also introduced a heterologous PDE, PA5295 from *Pseudomonas aeruginosa* [33], the cell type specific programs were restored (Figure 8C). By itself, or in combination with the constitutive DGC YdeH, PA5295 is unable to restore the bimodal program of *C. crescentus* (Figure 8B). Together, these results indicated that the correct cell-type specific control of either a DGC or a PDE is sufficient to maintain the bimodal developmental program of *C. crescentus*.

## Discussion

Many bacteria possess a network of multiple enzymes that produce and degrade the second messenger c-di-GMP. This preponderance of DGCs and PDEs raises two important questions. First, what is the functionality of all of these enzymes and what are the cellular processes controlled by their product? And second, are all c-di-GMP mediated processes within a cell intimately coordinated with each other through co-regulation by a common c-di-GMP pool or do insulated c-di-GMP modules exist? To address these questions this study systematically analyzed c-di-GMP dependent cellular processes and their dose-response curves in *C. crescentus*. For this, it was important to disentangle the global pleiotropic effects of c-di-GMP from DGC-specific and therefore source-dependent, c-di-GMP mediated traits.

To this end, we constructed a strain that is completely devoid of c-di-GMP and used it to restore distinct levels of the second messenger through the controlled expression of a heterologous DGC. A c-di-GMP free strain showed severe developmental defects and was unable to assemble polar organelles including holdfast, pili, flagellum and stalk. Furthermore, cells were strongly elongated with displaced division septa, lost their characteristic curvature and failed to undergo their typical, cell type specific density switch. While intervention with cell cycle progression has been shown to impinge on cell differentiation, cell cycle processes are largely independent of developmental processes in *C. crescentus*. It was therefore expected that c-di-GMP has a rather low impact on cell growth, despite these severe morphological changes of the c-di-GMP free strain. These findings are similar to what was reported in *Salmonella enteritidis*, which shows normal growth in the absence of c-di-GMP [34]. However, while long-term survival is compromised in a c-di-GMP free *Salmonella* strain, this was not the case in *C. crescentus* (data not shown). Likewise, when the c-di-GMP concentration was increased artificially up to 60-fold of the levels observed in the wild type, no effect on cell growth was observed. Thus, *C. crescentus* is either resistant to high levels of c-di-GMP or the concentrations that cause adverse effects on cell growth in other bacteria [6] could not be reached with the expression system used here.

### A role for c-di-GMP in the motile-sessile switch and beyond

Numerous studies have demonstrated that c-di-GMP negatively interferes with bacterial motility at different levels (e.g. [10], [15], [35]–[41]). This is also the case in *C. crescentus* where c-di-GMP obstructs motility in at least two different ways, by interfering with motor function in the swarmer cell [13], [15], [42] and by triggering flagellar ejection during the swarmer-to-stalked cell transition [13], [42]. One of the surprising findings of this study is that c-di-GMP also plays a critical role in flagellar assembly. This is in accordance with c-di-GMP being important for flagellar assembly in *Salmonella* [34] and argues that a specific c-di-GMP window defines both the lower and upper limits of motility in bacteria. It remains to be shown if such a bipartite role of c-di-GMP in flagellar-based motility regulation is a general phenomenon in bacteria. In *C. crescentus*, c-di-GMP mediated control may coordinate the assembly and function of the flagellar motor in time and space during the cell cycle. Dividing *Caulobacter* cells need to continuously re-orient their flagellar polarity. While the flagellum is removed from the incipient stalked pole during cell differentiation, it is reassembled in the predivisional cell at the pole opposite the stalk. At this stage of the cell cycle c-di-GMP levels are likely to be high enough to initiate the assembly of the structure and might also be high enough to obstruct its rotation until c-di-GMP levels drop after cell division releases a functional swarmer cell. This might assist the assembly process or facilitate a tight coordination between cell division and swimming behavior of the swarmer progeny. The latter is supported by the observation that mutants with lower levels of c-di-GMP show premature swimming behavior as predivisional cells [13], [14]. Since most of the c-di-GMP dependent processes in *C. crescentus* show inverted U-shaped dose-response curves, counteracting c-di-GMP dependent mechanisms - one activating and the other inhibitory - might represent a more general phenomenon.

Our study demonstrates that c-di-GMP not only orchestrates *C. crescentus* pole development, but also strongly contributes to cell morphology. While cells lacking c-di-GMP are straight and elongated, their characteristic length and crescentoid curvature is restored at intermediated levels of c-di-GMP and strongly increased at very high c-di-GMP concentrations. *C. crescentus* cell curvature depends on the intermediate filament Crescentin [43], while division septum placement depends on FtsZ and its organizer MipZ [44]. Although c-di-GMP has not been linked to elements of the cytoskeleton so far, it remains to be shown at which stage c-di-GMP interferes with these processes. Likewise, c-di-GMP is required for stalk elongation, a process that resembles cell elongation and that originated as an adaptation to surface growth in oligotrophic environments [13], [45], [46]. In the absence of c-di-GMP, stalks are not detectable in complex media. Under phosphate-limited conditions, stalk growth is partially rescued (data not shown), arguing that c-di-GMP regulation represents only one of several regulatory inputs into this process.

Finally, we find evidence that c-di-GMP influences the cell type specific expression of a capsule-like exopolysaccharide. The cdG^0^ strain is hypersensitive to phage φCR30, which docks to the surface exposed S-layer [23], [47]. This indicated that under these conditions a protective layer on the outside of the cell is missing leading to increased exposure of phage receptors. Likewise, differential cell density, a feature that is used to synchronize *C. crescentus* populations by density gradient centrifugation, is abolished in the cdG^0^ strain and restored upon *ydeH* expression with a dose-response curve indistinguishable from the corresponding dose –response curves of φCR30 sensitivity. While in the absence of c-di-GMP all cells show swarmer cell-like high density and φCR30 hypersensitivity, at high c-di-GMP concentrations all cells show a stalked cell-like low density and φCR30 resistance. In addition, cells without c-di-GMP form rough colonies on sugar-containing media in comparison to the mucoid wild type (data not shown). These phenotypes, but none of the other c-di-GMP dependent processes, hinge on a mobile genetic element (Figure 6A, B) that harbors several predicted glycosyl-transferases and other genes involved in carbohydrate metabolism and polymerization [23]. Together this argued that the two phenotypes are linked, and suggested that both processes are contingent on a surface exposed, cell type specific capsule-like exopolysaccharide. While such a structure has been described in *C. crescentus* [32], its cellular and biochemical properties remain to be characterized. Our studies predict that c-di-GMP regulation limits the expression of such a capsular structure to the sessile cell types, while it keeps the motile swarmer cell free of this extra surface layer, thereby lending this cell type its high density and phage sensitivity.

### Oscillation of c-di-GMP levels during the cell cycle

Efficient surface attachment of *C. crescentus* requires the concerted action of a rotating flagellum, type IV pili, and an adhesive holdfast [25], [26], [48]. We show here that the formation of all of these organelles depends on c-di-GMP. However, while flagellum and pili biogenesis are restored in the cdG^0^::*ydeH* strain at very low c-di-GMP levels, holdfast production and attachment only kick in at moderately high c-di-GMP concentrations. In principle, differential regulation of these processes can be explained by concentration differences of c-di-GMP in either time or space [1], [20]. A temporally oscillating global pool of c-di-GMP could elicit a graded response through the serial activation of processes with different activation thresholds for c-di-GMP. For example, the order of assembly of flagellum, pili and holdfast during the cell cycle could directly follow from temporal fluctuations of c-di-GMP levels, which are very low in the swarmer cell, peak at the swarmer-to-stalked cell transition and later drop to an intermediate level in the predivisional cell. In this model, c-di-GMP signaling specificity could be achieved through differences in receptor affinities as recently indicated with engineered receptor affinity mutants in *Salmonella enterica* serovar Typhimurium [5]. While these c-di-GMP binders could govern single specific phenotypes, we cannot exclude that one pathway regulates several traits. In the *C. crescentus* swarmer cell c-di-GMP levels are below 100 nM (Figure 7 and [20]). Consistently, we find that swarmer cell specific c-di-GMP regulated processes like motor function, pili expression and high cell buoyancy operate at low c-di-GMP levels. In contrast, stalked cell specific processes like holdfast and stalk biogenesis are not induced at such low concentrations, but coincide with a peak of c-di-GMP of about 275 nM during the motile-sessile transition. When considering the c-di-GMP dose response curves determined with a strain expressing a single heterologous DGC, these concentrations would not be sufficient to induce the motile-sessile switch. In a mixed culture of the cdG^0^::*ydeH* strain, the average c-di-GMP concentration might be a poor predictor of behavioral changes because the temporal c-di-GMP profile and thus the effective concentrations triggering specific phenotypes are unknown. Alternatively, it is possible that we underestimate the c-di-GMP concentration in synchronized cells. C-di-GMP measurements were not carried out in single cells but in populations of synchronized cells. Cells synchronized via density gradient centrifugation retain a certain degree of heterogeneity because of varying cell cycle length and different internal age of the harvested swarmer cells. Although our mathematical model adjusts for this heterogeneity, it makes assumptions regarding cell cycle length (normal distribution) and internal age at harvesting (uniform distribution) that might simplify reality. Also, because of limited temporal resolution (20-minute intervals) we might underestimate the effective maximum of the c-di-GMP concentration peak. Single cell based c-di-GMP measurements indicated that the second messenger reaches levels above 500 nM in the stalked cell [18], [20]. Interestingly, these FRET-based experiments failed to observe the c-di-GMP peak during the swarmer-to-stalked cell transition. This discrepancy is either due to the fact that FRET fails to accurately measure moderate c-di-GMP changes or because LC/MS based measurements reported previously [18] and in this study underestimate c-di-GMP levels specifically in the stalked and predivisional cell. The observation that different levels of c-di-GMP are required to initiate distinct processes in the stalked and predivisional cell (e.g. holdfast synthesis vs. flagellum assembly) argues that the c-di-GMP metabolism in the sessile cell types is more complex than anticipated by FRET measurements. The observed reduction of the second messenger concentration during the stalked cell phase also indicated that one or several phosphodiesterases are active during this stage of the cell cycle. The observation that a *cc0091* deletion has no effect on motility but strongly interferes with surface attachment is in agreement with a role for this PDE in the sessile stalked cell.

### Models of c-di-GMP specificity

C-di-GMP thresholds required to restore specific processes in the cdG^0^ strain largely correlate with the concentrations measured in different cell types *in vivo*. Although this is consistent with a global pool model where c-di-GMP mediates differential cell behavior primarily through different effector affinities, other observations indicated that c-di-GMP control goes beyond mere temporal variation. The global pool model predicts that c-di-GMP dependent processes that coincide during the cell cycle have similar activation thresholds and shared upstream components. This is not the case for developmental and cell cycle processes that coincide during the swarmer-to-stalked cell transition. Mutants lacking the DGCs PleD and DgcB fail to assemble a holdfast, while the coincident activation of the PopA pathway leading to replication initiation is not affected under these conditions [14], [15]. This strongly argues that these processes, although running in parallel, must have different activation thresholds for c-di-GMP. This, in turn implies that they are fueled by specific enzyme combinations. In such a scenario, distinct pathways might be individually regulated within spatially separated c-di-GMP environments, thereby providing more complex possibilities for regulatory fine-tuning. Compartmentalized pools could e.g. originate from a distinct arrangement of DGCs and/or PDEs, as observed for PleD, DgcB and PdeA in *C. crescentus* [14], [15] or in other bacteria [49], [50]. Alternatively, it could result from macromolecular complexes of DGCs and/or PDEs with their downstream effectors or from a similar arrangement of bifunctional trigger enzymes [51]. Although the finding that a heterologous source for c-di-GMP can complement all defects of the cdG^0^ strain could be interpreted in favor of a global pool model, phenotypic behavior and overall levels of c-di-GMP do not match the behavior of individual *dgc* and *pde* mutants. For example, although both motility and surface attachment are strongly affected in a *dgcB* mutant, the overall c-di-GMP levels show only a minor reduction as compared to the wild type. Similarly, compared to the overall changes in c-di-GMP content, mutants lacking PleD or PdeA show disproportionally strong behavioral changes. Finally, YdeH mediated rescue of c-di-GMP dependent processes in a cdG^0^ often remained below wild-type level and occurred at c-di-GMP concentrations higher than those observed in wild type. This suggested that specific c-di-GMP effectors might be more accessible for homologous DGCs and/or PDEs in the original signaling context, while in the cdG^0^::*ydeH* strain higher concentrations are required to ‘invade’ these signaling units and to compensate for the missing functions.

Taken together, our findings highlight the central importance of c-di-GMP in bacterial development and life-style decisions. They further indicate that both temporal gradients of a global c-di-GMP pool and insulated c-di-GMP micro-pools facilitate the complex coordination of development and cell cycle progression in *C. crescentus*.

## Materials and Methods

### Strains, plasmids and growth conditions

The bacterial strains and plasmids used in this study are listed in Table S1. *E. coli* strains were grown in Luria Broth (LB) medium at 37°C, supplemented with the appropriate antibiotic (solid/liquid media; in µg/ml: kanamycin 50/30, gentamycin 20/15, oxytetracycline 12.5/12.5). *C. crescentus* strains were grown in peptone yeast extract (PYE) or M2 minimal medium supplemented with 0.1% glucose (M2G) at 30°C. These media were also supplemented with the appropriate antibiotic (solid/liquid media; in µg/ml: kanamycin 20/5, gentamycin 5/0.5, oxytetracycline 5/2.5, nalidixic acid 20/n.a.) and the inducers vanillate (Van; 1 mM) and isopropyl 1-thio-b-D-galactopyranoside (IPTG; 31–1666 µM) where applicable. To solidify the medium, either 1.5% (regular growth plates) or 0.3% (motility plates) agar was added. The optical density of cultures were either determined individually using a photo-spectrometer at 660 nm (Genesys6, Thermo Spectronic, WI, USA) or in 96-well format using clear bottom plates (BD Falcon, NJ, USA) and a plate reader at 660 nm (Molecular Devices, CA, USA). The *E. coli* strain DH5α was used for cloning and plasmid propagation, while S17-1 was used for plasmid transfer in *C. crescentus* by conjugation as described by Ely et al. [52]. Plasmid construction is described in the supporting information (Text S1) and primers used for plasmid construction are listed in Table S2. Deletion mutants were generated by allelic exchange as described before [53]. In brief, the suicide plasmid pNPTS138 was used as plasmid backbone harboring two regions of homology that flank the gene of interest. After mobilization of the plasmid into *C. crescentus*, Kanamycin resistant first recombinants were selected, followed by a sucrose counter selection step. Sucrose resistant second recombinants were tested by PCR and confirmed by sequencing. To exclude the possibility that spontaneous mutations might have emerged during the construction of the cdG^0^ strain to suppress essential functions of c-di-GMP, the NA1000 cdG^0^ strain was re-sequenced. Comparison of ancestor and cdG^0^ mutant identified a total of five SNPs. Back-crossing experiments confirmed that none of the genetic changes had an influenced on the behavior of the cdG^0^ strain (data not shown).

### Microscopy

For microscopy, cells were harvested at mid-exponential phase (OD_660_∼0.3). Except for holdfast stains, all strains used for microscopy were generated in NA1000 background. For transmission electron microscopy (TEM), the cells were washed twice with water and absorbed to a glow-discharged, carbon-coated colloid film on a copper grid. The grids were then washed several times with deionized water and negative stained with 0.75% (w/v) uranyl formate. In case cells were infected with φCbK, the phage was added after the first washing steps 15 min before cells were fixed with an aqueous formaldehyde solution (1%). Cells were examined with a Hitachi 7000, 100 kV instrument.

For light microscopy, cells were placed on agarose pads (1% in water, Sigma, USA). Fluorescence, phase contrast (PH), and differential interference contrast (DIC) pictures were taken with a DeltaVision Core microscope (Applied Precision, USA) equipped with an UPlanSApo 1003/1.40 Oil objective (Olympus, Japan) and a coolSNAP HQ-2 CCD camera (Photometrics, USA). Images were processed with softWoRx version 5.0.0 (Applied Precision, USA) and Photoshop CS6 (Adobe, USA) software. Cellular dimensions, single cell fluorescence, and number of foci were analyzed using MicrobeTracker version 0.931 [54].

### Holdfast stain

To visualize the holdfast, cells (CB15 or NA1000 *hfsA* ^+^ background) in mid-exponential growth phase were stained with Oregon Green-conjugated wheat-germ agglutinin (0.2 mg/ml, Invitrogen, USA), washed twice with water and visualized by fluorescence microscopy. To quantify the holdfast production, images of stained cells were segmented by setting a threshold that removes background signal. Individual stained holdfasts were identified in these bitmap images by using the “analyze particle” tool from imageJ [55] with selection for >0.7 circularity and >2 pixel size. The intensities of holdfast stains were then quantified in the identified regions as measure for holdfast production and analyzed in R [56].

### Density gradient centrifugation

For small-scale density gradient centrifugations, cells were grown in PYE medium until they reached mid-exponential growth phase. A 20× staining solution (0.1% Coomassie brilliant blue R in 40% methanol; 10% acetic acid; 50% water) was added to optimize the visibility of high and low density bands and incubated for 10 min at room temperature. Cells were washed twice with cold synchrony phosphate buffer (12.25 mM Na_2_HPO_4_; 7.75 mM KH_2_PO_4_) and resuspended in cold 33% Ludox (in synchrony phosphate buffer). After 45 min centrifugation at 9000×g at 4°C, pictures were taken (Nikon Coolpix 990) with back light illumination to document the band distribution.

Big scale density gradient centrifugations for isolating swarmer cells were performed in M2G medium as described before [53]. If necessary, inducible promoters were spiked three hours prior to the density gradient centrifugation. After release of isolated swarmer cells in fresh medium, samples for light microscopy and immunoblot analysis were taken every 20 min for a total of 160 min. All density gradient centrifugation experiments were performed with NA1000 derived strains.

### β-galactosidase assays

To determine the β-galactosidase activities of promoter *lacZ* fusions, strains carrying the reported constructs were grown to mid-exponential growth phase in M2G-medium containing tetracycline. The cells were permeabilized with chloroform and SDS and assayed in triplicate as described by Miller et al. [57].

### SDS-PAGE and immunoblot analysis

*C. crescentus* cells were harvested by centrifugation at ∼20,000×g at 4°C. Pellets were resuspended in SDS loading buffer (50 mM Tris-HCl (pH 6.8); 2% sodium dodecyl sulfate (SDS); 10 mM dithiothreitol (DTT); 10% glycerol) and normalized for the optical density of the culture. Total protein lysates were separated by 12.5% SDS-polyacrylamide gel electrophoresis (PAGE) and transferred to PVDF-membranes (Immobilon-P, Millipore, MA, USA). Proteins were detected using specific polyclonal antibodies (anti-CtrA 1∶5,000; anti-McpA 1∶10,000; anti-CcrM 1∶10,000; anti-PdeA 1∶1,000) and polyclonal anti-rabbit secondary antibodies conjugated to horseradish peroxidase (1∶10000; Dako, Denmark). Flag-tagged YdeH was detected by using M2-antibodies (1∶10000; Invitrogen, USA) and anti-mouse secondary antibodies conjugated to horseradish peroxidase (1∶10000; Dako, Denmark). After incubation with ECL chemiluminescent substrate (Perkin Elmer, USA), Super RX X-ray films (Fuji, Japan) were used to detect luminescence. Band intensities were quantified using the integrated density tool from imageJ after scanning the exposed X-ray films.

### Attachment and motility assay

Motility of cells was determined on semi-solid PYE plates and surface attachment was quantified in 96-well polystyrene plates in PYE as described before [14], [25]. Attachment assays were performed with cells derived from CB15 or NA1000 *hfsA* ^+^ and grown for 24 h before the biofilm was quantified. Motility assays in Figure 1, 2, 5 and S7 show mutants created in CB15 background, Figure 6 and S10 were performed with strains created in NA1000 or NA1000 *hfsA* ^+^ background.

### Phage sensitivity and general transduction

To determine the sensitivity of *C. crescentus* cells to φCbK and φCR30, cells were grown to mid-exponential phase, embedded in molten PYE containing 0.45% agar at 37°C and spread on PYE plates. After the agar had solidified, a 1∶10 serial dilution of the appropriate phage was spotted on the bacterial lawn. The plates were incubated for 48 h at 30°C and pictures were taken (Nikon Coolpix 990) using an indirect illumination box [58]. For the transduction of wild-type alleles of *cc0091*, *cc1086*, and *pdeA*, φCR30 lysates were prepared on wild-type NA1000 cells carrying a kanamycin marker in the vicinity of the gene of interest (CMS0, CMS12, and CMS37, respectively) [59]. These lysates were used to infect the recipient strains. After selection for kanamycin, the presence of the correct allele was determined by PCR. Lysate preparation and general transduction were performed as described before [59].

### c-di-GMP extraction and quantification

C-di-GMP was extracted from NA1000 and NA1000 derived strains and quantified by liquid chromatography-tandem mass spectrometry as described previously [31]. The average intracellular concentration was determined by normalizing the c-di-GMP measurements to the total bacterial volume as determined by the median cell volume and the CFU per OD_660_.

### Genome sequencing and mapping of mutations

Genomic DNA was extracted from NA1000 cdG^0^ and the parental strain (NA1000jenal) using standard guanidium thiocyanate extraction and isopropanol/ethanol precipitation. The DNA was sequenced at Fasteris (Switzerland) on the Genome Analyzer GAIIx platform generating 2×38 bp paired end reads. These data were mapped on the NA1000 reference genome (GenBank accession CP001340; [23]) using VAAL [60]. Sanger sequencing was used to confirm polymorphisms.

### Modeling of c-di-GMP fluctuations during the cell cycle of single cells

Synchronized populations of *C. crescentus* cells retain a certain degree of heterogeneity. To infer stage-specific c-di-GMP contents we employed a mathematical model that extracts these data from population measurements. The first step was to determine the exact composition of the cell population at any given time point. We considered two sources of heterogeneity in synchronized populations of *C. crescentus*: i) variations in cell cycle length and ii) variations in age of newborn swarmer cells harvested by density gradient centrifugation. We followed the individual (“internal”) age of each cell in a virtual bacterial population, where cells with a characteristic cell cycle length *t_C_* divide asymmetrically into two daughter cells with the internal age 0 (swarmer cell) and *t_S_* (stalked cell) (Figure S11C, D). The cell cycle length was determined from OD_660_ measurements in the cultures in which c-di-GMP was quantified (*t_C_* = 137 min). We assume that the length of G1 (swarmer cell) and S+G2 (stalked and predivisional cell) are ¼ and ¾ of the full cell cycle length, such that *t_S_* = 0.25 *t_C_*. Additionally, we make the assumption that stalked cells have the same c-di-GMP content independent of their origin, e.g. differentiated swarmer cells or newborn stalked cell originating from cell division. The cell cycle length of individual bacteria in the population was assumed to follow a normal distribution with a standard deviation as determined from previously published data (+/−35%) [61].

To calculate c-di-GMP concentrations in individual cells from the population measurements, we grouped the bacterial population in seven 20-min intervals *i* and solved the resulting system of equations for the measured c-di-GMP content:
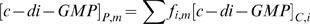
with *m* describing the timepoint of the measurement (0–180 min), *[c-d-GMP]_P,m_* the average c-di-GMP content in a *C. crescentus* population at this timepoint (Figure S11A), *f_i,m_* the fraction of cells at the timepoint *m* that have an internal age in the interval *i* and *[c-d-GMP]_C,i_* as the average c-di-GMP concentration in cells in the age interval *i*. All calculations were done in R.

## Supporting Information

Figure S1 Domain organization of *C. crescentus* GGDEF and EAL domain proteins. This figure illustrates the domain organization of all known GGDEF and EAL domain proteins from *C. crescentus* as predicted by SMART (www.smart.embl-heidelberg.de). GGDEF domains are shown in red while EAL domains are highlighted in blue. Receiver (REC), coiled-coil (CC), Per-Arnt-Sim (PAS), MHYT and Chase4 domains are depicted in light grey. Black vertical bars represent predicted trans-membrane domains. The size of each illustration reflects the length of the protein/domain in amino acids. The name of the protein and corresponding gene number (CC_) is given on the right to the illustration and highlighted in red for known DGCs, blue for known PDEs and dark grey for enzymatically inactive proteins.
(TIF)

Figure S2 Controlled *ydeH* expression tunes c-di-GMP over a wide concentration range. The c-di-GMP concentration was experimentally determined in the cdG^0^ strain background expressing *ydeH* from the chromosome (green dots), a low copy (blue dots) or a medium copy number plasmid (purple dots) at different IPTG concentrations. Levels of c-di-GMP (µM) of wild type (orange dots) and the cdG^0^ strain (red dots) carrying a control plasmid are indicated for comparison. The dotted line indicates the average c-di-GMP concentration in the wild type without IPTG from nine measurements. Concentrations were calculated as described in Materials and Methods.
(TIF)

Figure S3 The c-di-GMP production is required to complement the cdG^0^ strain. Surface attachment (black bars) and colony size on motility agar plates (grey bars) of *dgcB* or *pleD* mutants and the cdG^0^ strain expressing wild-type DGCs or active site mutants, respectively. A) Strains expressing DgcB wild-type (*dgcB* ^+^) or a DgcB active site mutant (*dgcB*_E261Q_) from the chromosomal *dgcB* locus. B) Strains expressing PleD (pPleD) or its active site mutant (pPleD_GG368DE_) from expression plasmids. Strains without indicated plasmid carry empty control plasmids (pSA129). Each bar represents the mean of at least ten experiments; the error bars represent the standard deviation; the dotted line indicates the wild-type behavior. Active site mutants were expressed at similar level as wild-type proteins (data not shown).
(TIF)

Figure S4 Expression systems used to control the cellular c-di-GMP concentration. A schematic representation of the chromosomal and plasmid-based YdeH expression systems used in this study.
(TIF)

Figure S5 The IPTG-inducible expression system can be used for discrete and uniform YdeH expression. A) A *lac* promoter-based expression system allows tunable expression of the *E. coli* diguanylate cyclase YdeH in the c-di-GMP free strain. The inducible *ydeH* gene is fused to a *flag*-tag and integrated into the chromosome (cdG^0^::*ydeH*) or introduced on an RK2-based low copy number (pSA280) or a pBBR-based medium copy number (pTB4) plasmid. The expression of YdeH is induced by different concentrations of IPTG and monitored by immunoblots with Flag-specific antibodies. A representative immunoblot is shown. The band intensities were quantified (IntDen) and are indicated as arbitrary units. The bars represent the mean and the error bars indicate the standard deviation of three independent experiments. B) YdeH driven from Plac does not fluctuate during the cell cycle. A population of swarmer cells from cdG^0^::*ydeH* was induced with 555 uM IPTG and followed through one cell cycle. Samples were taken in 20 min intervals and analyzed by immunoblot with antibodies directed against the Flag-tag. A representative immunoblot is shown. Cell-cycle progression is shown schematically above the blot. The band intensities at each time point were quantified (IntDen) and the mean of three experiments is shown in arbitrary units. Error bars represent the standard deviation. C) YdeH is homogeneously expressed and distributed on single cell level. A plasmid-borne copy of YdeH fused to GFP under control of the *lac* promoter was induced in wild-type *C. crescentus* by addition of 62 uM IPTG to the growth medium. After 3 h of induction, the fusion protein was visualized by fluorescent microscopy. A representative fluorescent and the corresponding phase contrast image are shown. The fluorescent intensity of the GFP signal was quantified in more than 1600 individual cells and normalized to the strongest signal. The distribution of these intensities is shown in a histogram. Furthermore, the distribution of the fluorescent signal within the cell was analyzed and is given as number of foci within a cell.
(TIF)

Figure S6 Determination of the *C. crescentus* cell volume. A–C) The volume, length and width of an average *C. crescentus* cells. Micrographs of an exponential wild type culture were taken and the distribution of the volume (A), length (B) and width (C) of more than 4000 cells was determined. D) The number of viable cells in an exponentially growing liquid culture. The colony forming units (CFUs) of a fixed volume of wild-type *C. cresentus* cultures with different optical densities (OD) were determined and plotted against each other. The solid line indicates the linear regression. The coefficient of determination (R^2^), the p-value (p), and the number of CFUs per 1 ml of an OD_660_ 1 culture are given in the graph.
(TIF)

Figure S7 *In vivo* dose-response curves for c-di-GMP dependent processes. Cell morphology (A), phage sensitivity (B, C) and cell type-specific cell density (D) was recorded as a function of varying c-di-GMP concentration in a cdG^0^ strain expressing YdeH, a heterologous DGC. YdeH expression conditions and resulting c-di-GMP concentration are taken from Figure S2. Table 2 summarizes these data and Figure 4 shows the same data at key c-di-GMP levels. A) *C. crescentus* cell length and morphology is controlled by c-di-GMP. Light micrographs of cells with increasing concentrations of c-di-GMP are shown. Wild-type cells carrying a control plasmid are shown for comparison. B–C) Interference with phage sensitivity at low and high c-di-GMP concentrations. Plaque assays are shown for lawns of cells with increasing concentrations of c-di-GMP with 1∶10 serial dilutions of the pili specific phage φCbk (B) and the S-layer specific phage φCR30 (C). D) Cell density is c-di-GMP dependent. *C. crescentus* cells with increasing intracellular c-di-GMP concentrations were separated by density gradient centrifugation. The resulting low- and high-density bands are highlighted by arrows.
(TIF)

Figure S8 IPTG does not influence c-di-GMP regulated processes. Wild type and cdG^0^ were grown in the presence of different concentrations of IPTG and tested for motility (A), surface attachment (B), morphology (C), φCbk (D) and φCR30 (E) phage sensitivity, as well as cell density (F). Motility and attachment assays were repeated five or eight times, respectively. The bars indicate the mean; error bars represent the standard deviation; the dotted line highlights wild-type behavior. G) Flagellar protein biosynthesis is not down regulated at high c-di-GMP concentrations. Immunoblots quantifying the FlgH expression levels in the wild type and the cdG^0^. In addition to a control plasmid, the latter either contained a chromosomal or plasmid-born copy of YdeH under control of the inducible lac promoter. All strains were induced with different IPTG concentrations. H) Cells with high c-di-GMP levels are flagellated. TEM pictures of negative stained NA1000 (wt) and cdG^0^ pTB4 grown in minimal medium containing 555 uM IPTG are shown. The flagellum attached to stalkes is highlighted by arrows. The scale bar is 1000 nm or 2000 nm, respectively.
(TIF)

Figure S9 The intracellular c-di-GMP concentration does not influence cell growth. A) IPTG does not influence the growth of *C. crescentus*. The growth curves of the wild type carrying the pBBR based, lac promoter containing control plasmid pSRK-Km were determined in complex medium containing different concentrations of IPTG by following the optical density at 660 nm (OD_660_) over time. B) The growth curves of different YdeH expression strains were recorded at different concentrations of the YdeH inducer IPTG in complex medium containing kanamycin (PYE-Kan). These were compared to the wild type and the cdG^0^ strain carrying a control plasmid in the absence of inducer. All growth experiments were performed with NA1000 derived strains.
(TIF)

Figure S10 Cell density and φCR30 phage sensitivity are regulated by c-di-GMP via a mobile genetic element. Same as in Figure 6, but with all control strains. The wild type, the mobile genetic element mutant (ΔMGE) and the cdG^0^ strain are shown, either carrying a YdeH overexpression construct (pTB4) or the empty vector backbone (pSRK-Km), both either with full IPTG induction or without inducer. These were tested for the cell density switch (A), the φCR30 (B) and φCbK (C) sensitivity, motility (D), surface attachment (E) and YdeH-3×Flag expression (F).
(TIF)

Figure S11 C-di-GMP fluctuations in synchronized *C. crescentus* populations. A) Quantification of the average intracellular c-di-GMP concentration of a synchronized *C. cresentus* population. Swarmer cells of three independent *C. crescentus* wild type cultures were harvested by density gradient centrifugation and followed for 180 min. Every 20 min the c-di-GMP concentration of the population is determined and the average intracellular c-di-GMP concentration is given in nM. The dotted line indicates the average c-di-GMP concentration in a mixed wild type population. B) Model of the internal age distribution of a synchronized *C. crescentus* population. Starting from a swarmer cell population, a snapshot of the internal age distribution is depicted every 20 min for a total of 160 min. C–D) Model of a *C. crescentus* population starting form a single cell. On the X-axis the total time of growth is given in minutes. On the Y-axis, either the number of cells (C) or the internal time in minutes (D) is given. The internal time starts at zero. When it reaches 137 minutes (*t_C_*) cells divide and the internal time is reset in both progeny to 0 minutes (swarmer cells) or ∼35 minutes (stalked cells, *t_S_*), respectively. Individual progeny are distinguishable by the color code and line structure.
(TIF)

Table S1 Strains, phages and plasmids used in this study.
(PDF)

Table S2 Primers used for plasmid construction.
(PDF)

Text S1 Construction of plasmids.
(PDF)
