# Supplementary figures and images for: Bi-modal Distribution of the Second Messenger c-di-GMP Controls Cell Fate and Asymmetry during the Caulobacter Cell Cycle
Source: PLoS Genet. 2013 Sep 5;9(9):e1003744. doi: 10.1371/journal.pgen.1003744 (PMC3764195; doi:10.1371/journal.pgen.1003744)

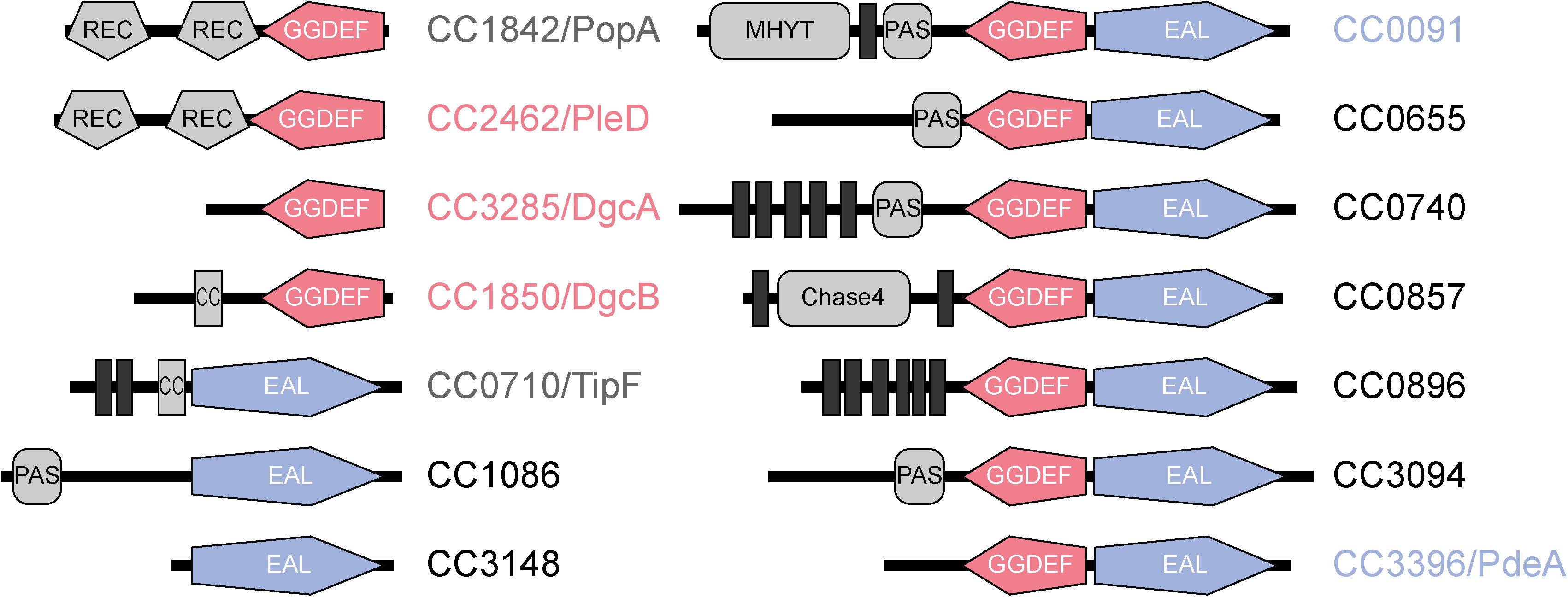

Supplement: Figure S1 — Domain organization of C. crescentus GGDEF and EAL domain proteins. This figure illustrates the domain organization of all known GGDEF and EAL domain proteins from C. crescentus as predicted by SMART (www.smart.embl-heidelberg.de). GGDEF domains are shown in red while EAL domains are highlighted in blue. Receiver (REC), coiled-coil (CC), Per-Arnt-Sim (PAS), MHYT and Chase4 domains are depicted in light grey. Black vertical bars represent predicted trans-membrane domains. The size of each illustration reflects the length of the protein/domain in amino acids. The name of the protein and corresponding gene number (CC_) is given on the right to the illustration and highlighted in red for known DGCs, blue for known PDEs and dark grey for enzymatically inactive proteins. (TIF) [file pgen.1003744.s001.tif]

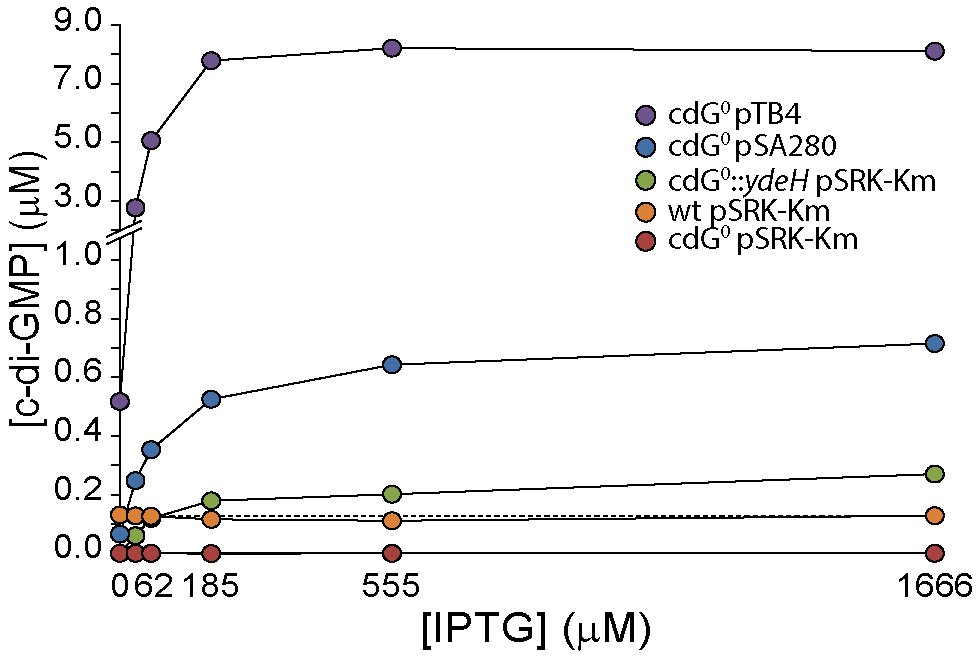

Supplement: Figure S2 — Controlled ydeH expression tunes c-di-GMP over a wide concentration range. The c-di-GMP concentration was experimentally determined in the cdG0 strain background expressing ydeH from the chromosome (green dots), a low copy (blue dots) or a medium copy number plasmid (purple dots) at different IPTG concentrations. Levels of c-di-GMP (µM) of wild type (orange dots) and the cdG0 strain (red dots) carrying a control plasmid are indicated for comparison. The dotted line indicates the average c-di-GMP concentration in the wild type without IPTG from nine measurements. Concentrations were calculated as described in Materials and Methods. (TIF) [file pgen.1003744.s002.tif]

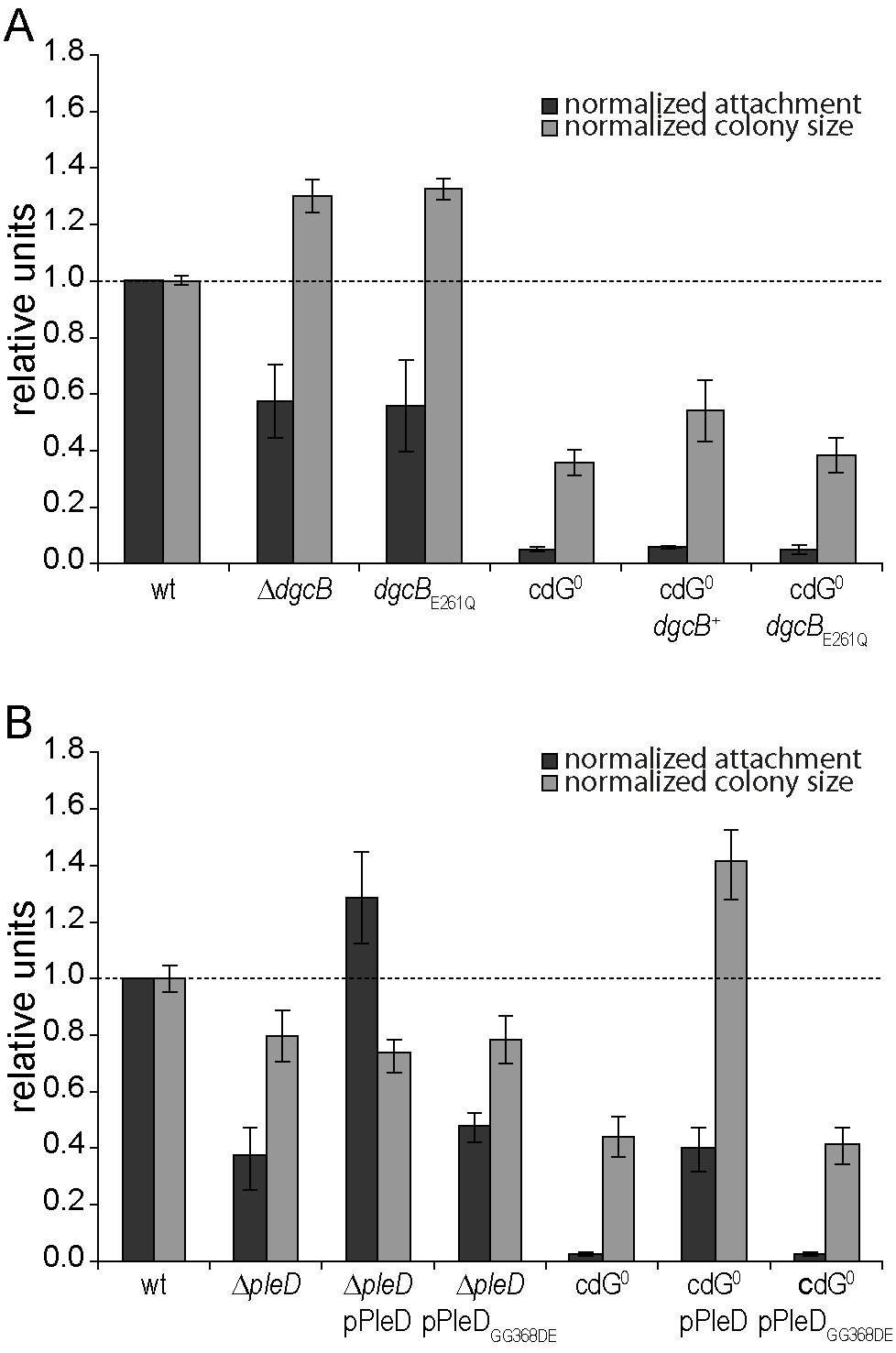

Supplement: Figure S3 — The c-di-GMP production is required to complement the cdG0 strain. Surface attachment (black bars) and colony size on motility agar plates (grey bars) of dgcB or pleD mutants and the cdG0 strain expressing wild-type DGCs or active site mutants, respectively. A) Strains expressing DgcB wild-type (dgcB +) or a DgcB active site mutant (dgcB E261Q) from the chromosomal dgcB locus. B) Strains expressing PleD (pPleD) or its active site mutant (pPleDGG368DE) from expression plasmids. Strains without indicated plasmid carry empty control plasmids (pSA129). Each bar represents the mean of at least ten experiments; the error bars represent the standard deviation; the dotted line indicates the wild-type behavior. Active site mutants were expressed at similar level as wild-type proteins (data not shown). (TIF) [file pgen.1003744.s003.tif]

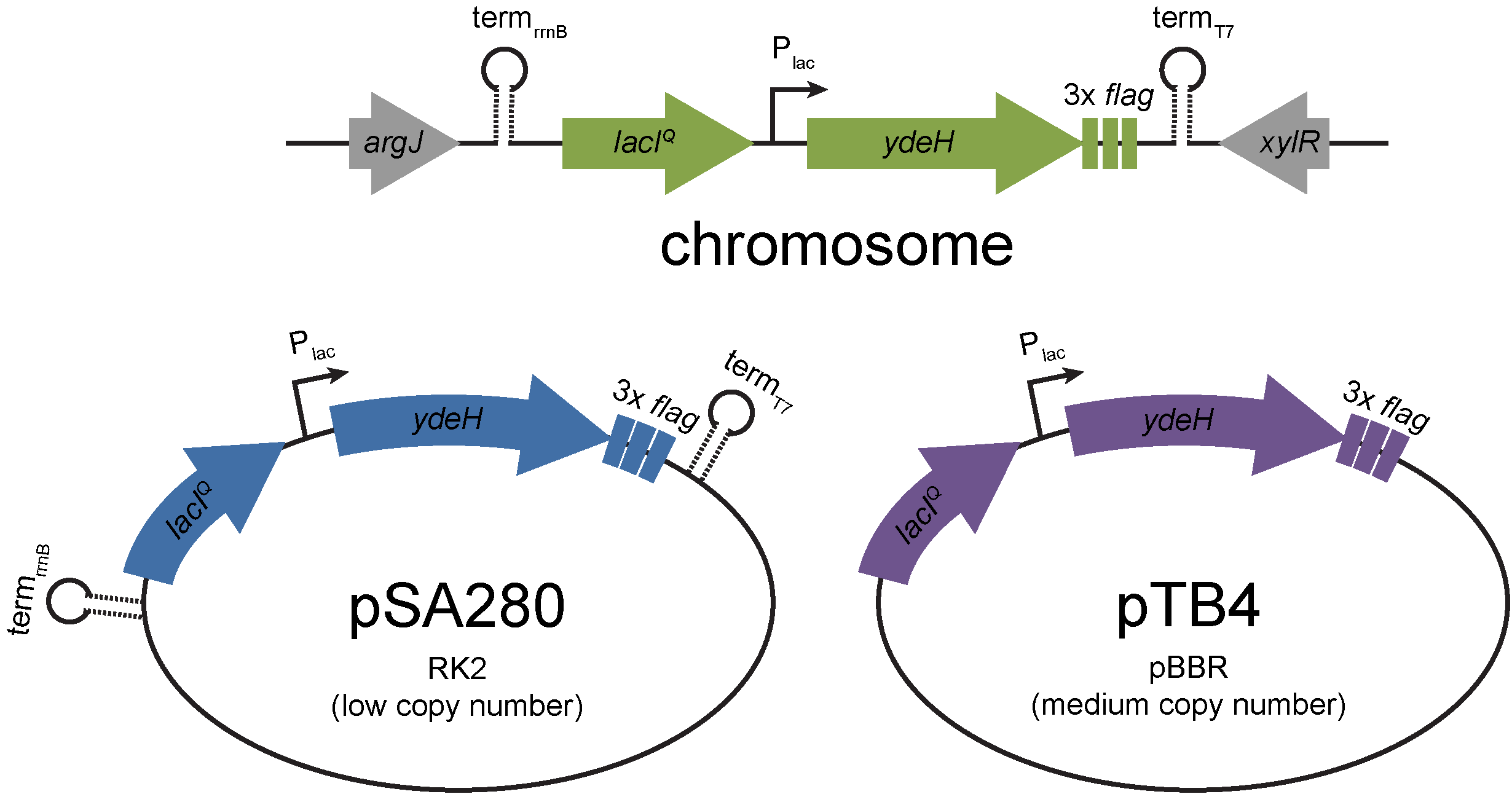

Supplement: Figure S4 — Expression systems used to control the cellular c-di-GMP concentration. A schematic representation of the chromosomal and plasmid-based YdeH expression systems used in this study. (TIF) [file pgen.1003744.s004.tif]

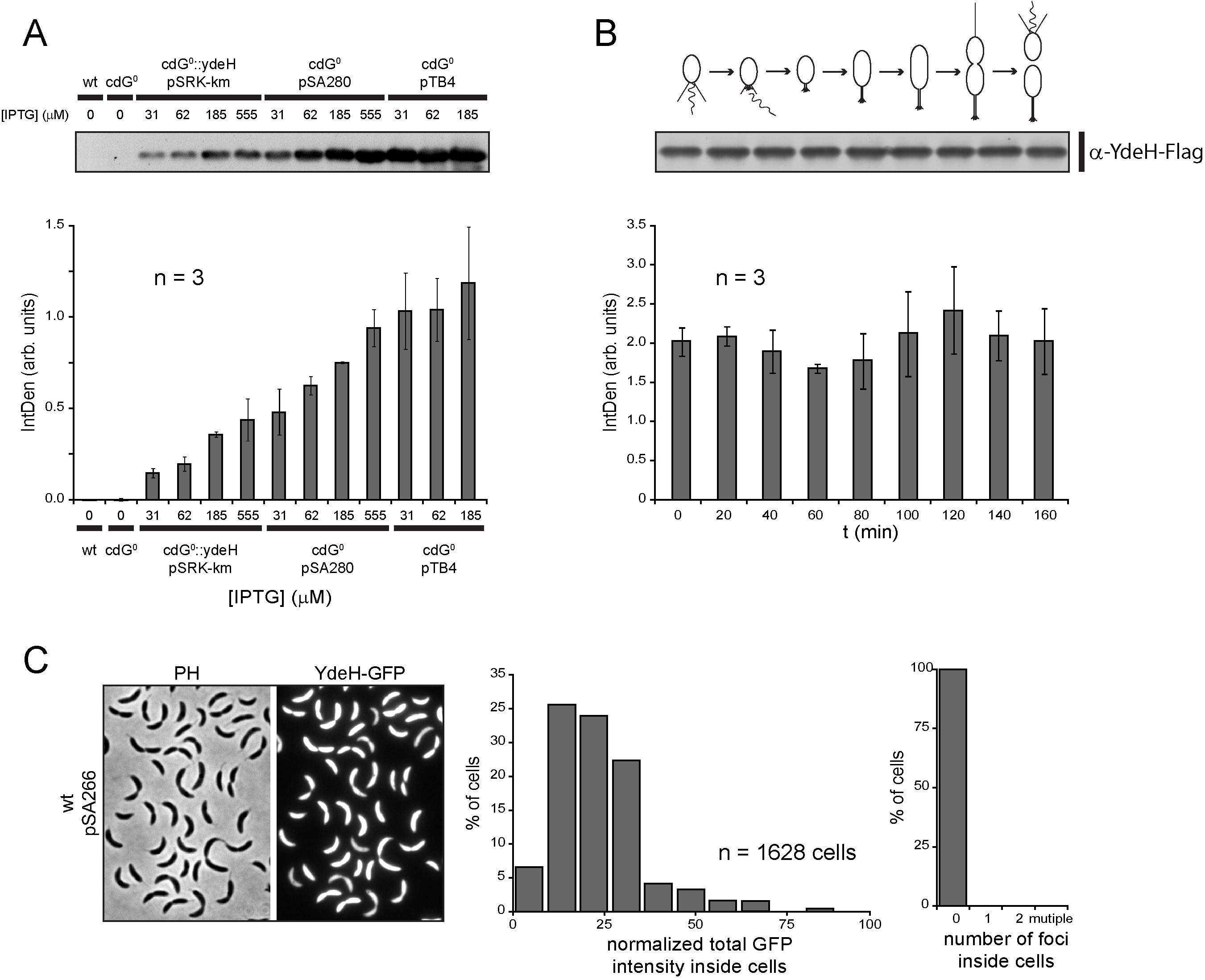

Supplement: Figure S5 — The IPTG-inducible expression system can be used for discrete and uniform YdeH expression. A) A lac promoter-based expression system allows tunable expression of the E. coli diguanylate cyclase YdeH in the c-di-GMP free strain. The inducible ydeH gene is fused to a flag-tag and integrated into the chromosome (cdG0::ydeH) or introduced on an RK2-based low copy number (pSA280) or a pBBR-based medium copy number (pTB4) plasmid. The expression of YdeH is induced by different concentrations of IPTG and monitored by immunoblots with Flag-specific antibodies. A representative immunoblot is shown. The band intensities were quantified (IntDen) and are indicated as arbitrary units. The bars represent the mean and the error bars indicate the standard deviation of three independent experiments. B) YdeH driven from Plac does not fluctuate during the cell cycle. A population of swarmer cells from cdG0::ydeH was induced with 555 uM IPTG and followed through one cell cycle. Samples were taken in 20 min intervals and analyzed by immunoblot with antibodies directed against the Flag-tag. A representative immunoblot is shown. Cell-cycle progression is shown schematically above the blot. The band intensities at each time point were quantified (IntDen) and the mean of three experiments is shown in arbitrary units. Error bars represent the standard deviation. C) YdeH is homogeneously expressed and distributed on single cell level. A plasmid-borne copy of YdeH fused to GFP under control of the lac promoter was induced in wild-type C. crescentus by addition of 62 uM IPTG to the growth medium. After 3 h of induction, the fusion protein was visualized by fluorescent microscopy. A representative fluorescent and the corresponding phase contrast image are shown. The fluorescent intensity of the GFP signal was quantified in more than 1600 individual cells and normalized to the strongest signal. The distribution of these intensities is shown in a histogram. Furthermore, the distribution of the flu [file pgen.1003744.s005.tif]

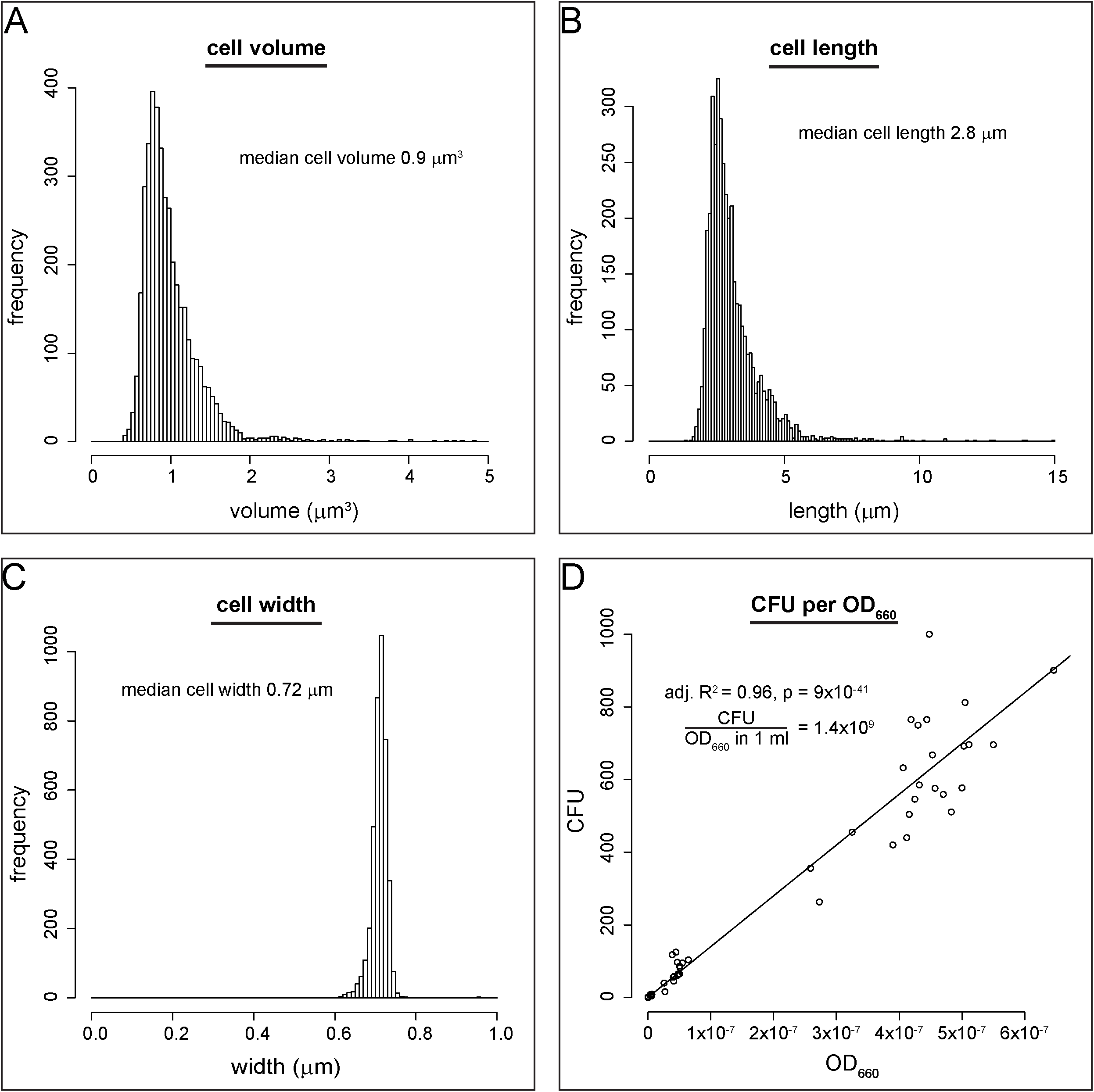

Supplement: Figure S6 — Determination of the C. crescentus cell volume. A–C) The volume, length and width of an average C. crescentus cells. Micrographs of an exponential wild type culture were taken and the distribution of the volume (A), length (B) and width (C) of more than 4000 cells was determined. D) The number of viable cells in an exponentially growing liquid culture. The colony forming units (CFUs) of a fixed volume of wild-type C. cresentus cultures with different optical densities (OD) were determined and plotted against each other. The solid line indicates the linear regression. The coefficient of determination (R2), the p-value (p), and the number of CFUs per 1 ml of an OD660 1 culture are given in the graph. (TIF) [file pgen.1003744.s006.tif]

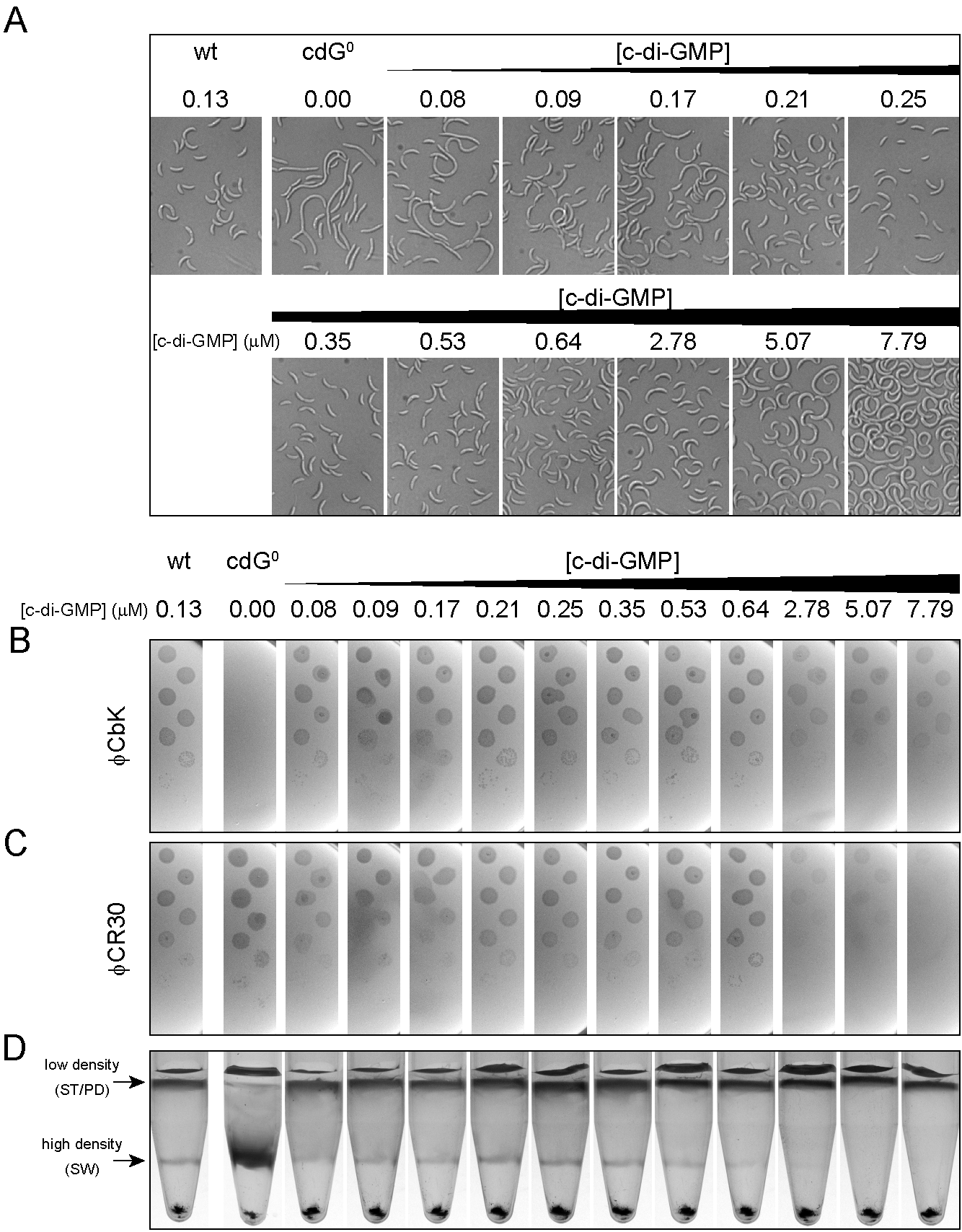

Supplement: Figure S7 — In vivo dose-response curves for c-di-GMP dependent processes. Cell morphology (A), phage sensitivity (B, C) and cell type-specific cell density (D) was recorded as a function of varying c-di-GMP concentration in a cdG0 strain expressing YdeH, a heterologous DGC. YdeH expression conditions and resulting c-di-GMP concentration are taken from Figure S2. Table 2 summarizes these data and Figure 4 shows the same data at key c-di-GMP levels. A) C. crescentus cell length and morphology is controlled by c-di-GMP. Light micrographs of cells with increasing concentrations of c-di-GMP are shown. Wild-type cells carrying a control plasmid are shown for comparison. B–C) Interference with phage sensitivity at low and high c-di-GMP concentrations. Plaque assays are shown for lawns of cells with increasing concentrations of c-di-GMP with 1∶10 serial dilutions of the pili specific phage φCbk (B) and the S-layer specific phage φCR30 (C). D) Cell density is c-di-GMP dependent. C. crescentus cells with increasing intracellular c-di-GMP concentrations were separated by density gradient centrifugation. The resulting low- and high-density bands are highlighted by arrows. (TIF) [file pgen.1003744.s007.tif]

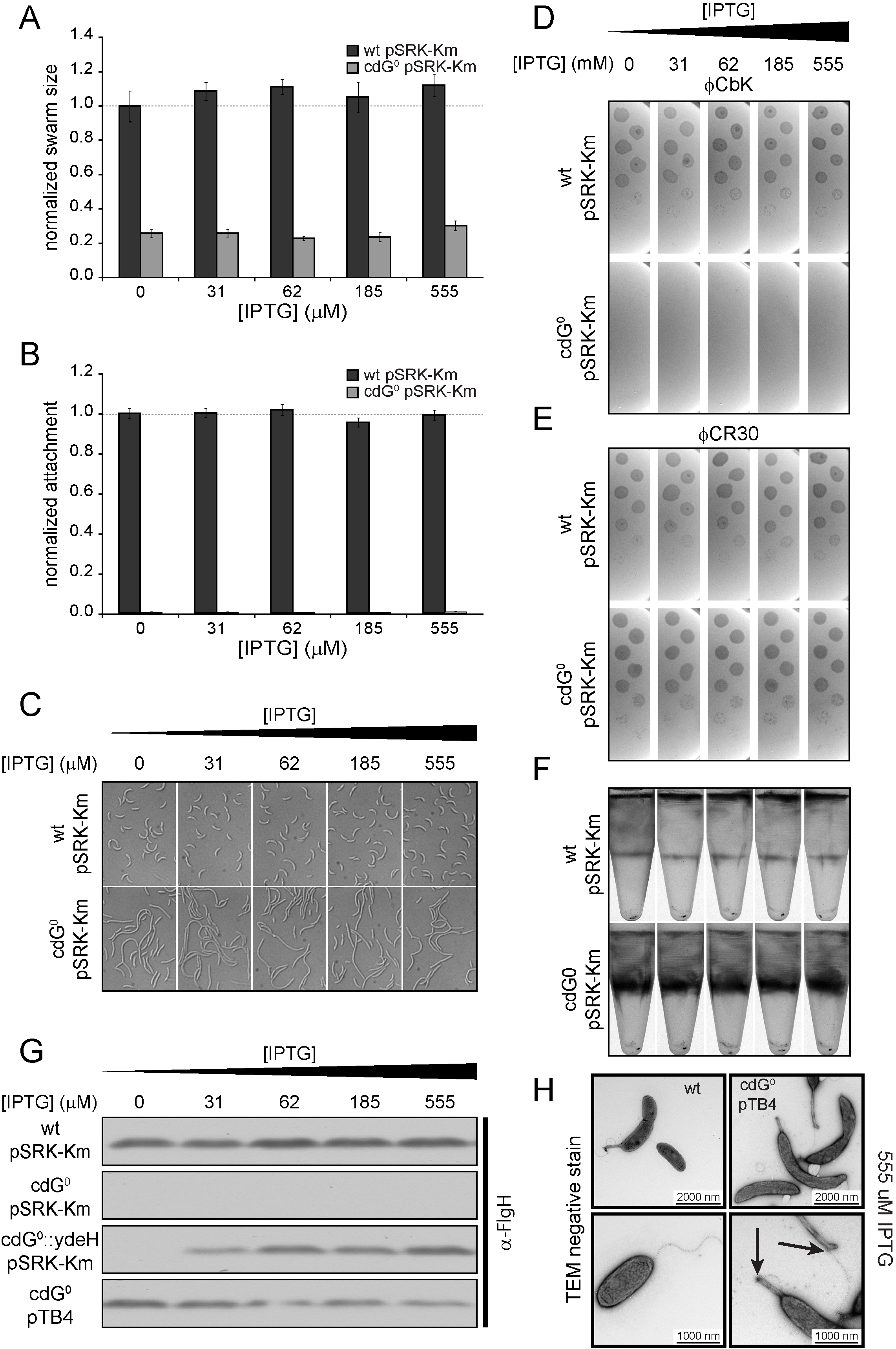

Supplement: Figure S8 — IPTG does not influence c-di-GMP regulated processes. Wild type and cdG0 were grown in the presence of different concentrations of IPTG and tested for motility (A), surface attachment (B), morphology (C), φCbk (D) and φCR30 (E) phage sensitivity, as well as cell density (F). Motility and attachment assays were repeated five or eight times, respectively. The bars indicate the mean; error bars represent the standard deviation; the dotted line highlights wild-type behavior. G) Flagellar protein biosynthesis is not down regulated at high c-di-GMP concentrations. Immunoblots quantifying the FlgH expression levels in the wild type and the cdG0. In addition to a control plasmid, the latter either contained a chromosomal or plasmid-born copy of YdeH under control of the inducible lac promoter. All strains were induced with different IPTG concentrations. H) Cells with high c-di-GMP levels are flagellated. TEM pictures of negative stained NA1000 (wt) and cdG0 pTB4 grown in minimal medium containing 555 uM IPTG are shown. The flagellum attached to stalkes is highlighted by arrows. The scale bar is 1000 nm or 2000 nm, respectively. (TIF) [file pgen.1003744.s008.tif]

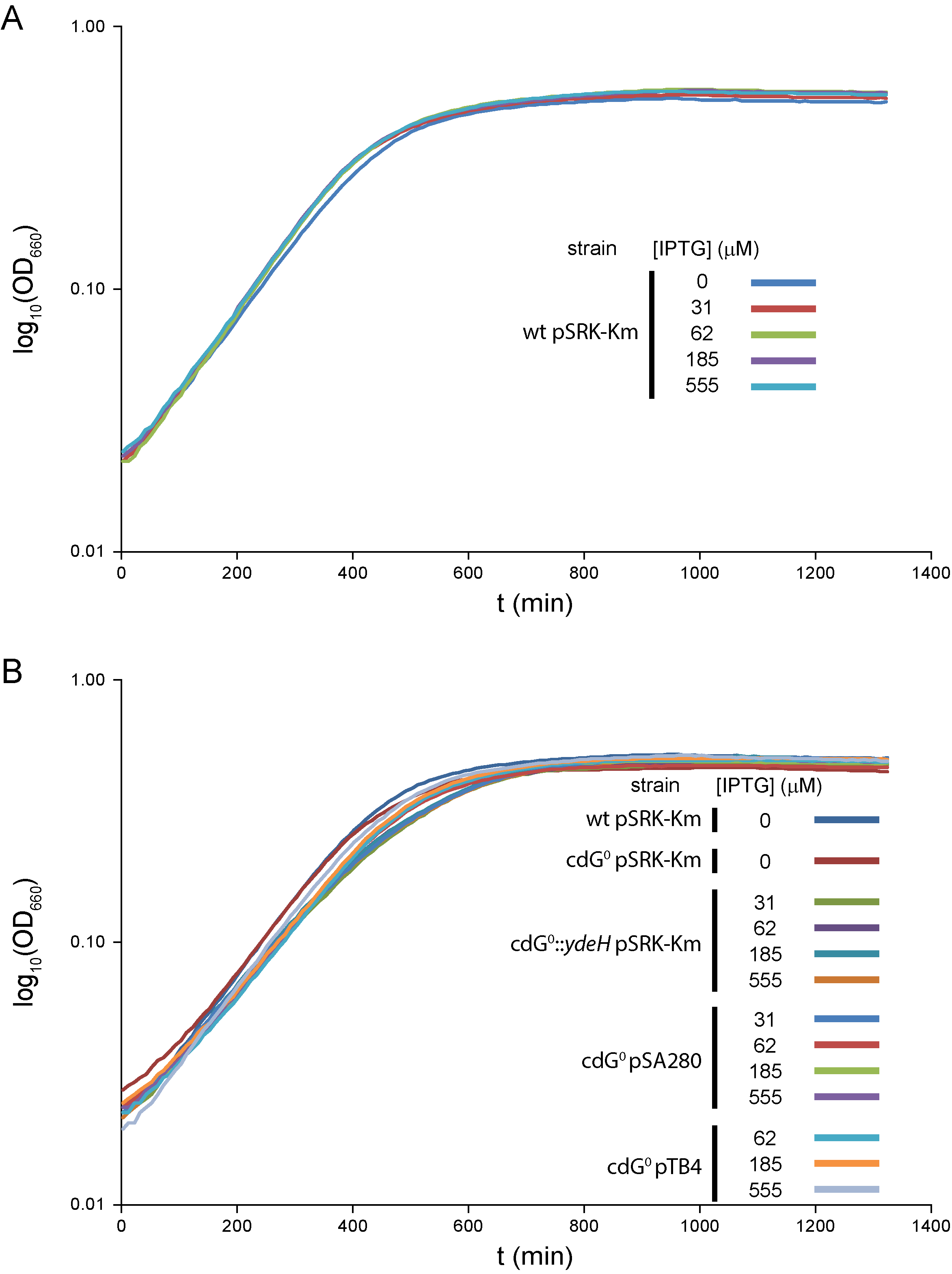

Supplement: Figure S9 — The intracellular c-di-GMP concentration does not influence cell growth. A) IPTG does not influence the growth of C. crescentus. The growth curves of the wild type carrying the pBBR based, lac promoter containing control plasmid pSRK-Km were determined in complex medium containing different concentrations of IPTG by following the optical density at 660 nm (OD660) over time. B) The growth curves of different YdeH expression strains were recorded at different concentrations of the YdeH inducer IPTG in complex medium containing kanamycin (PYE-Kan). These were compared to the wild type and the cdG0 strain carrying a control plasmid in the absence of inducer. All growth experiments were performed with NA1000 derived strains. (TIF) [file pgen.1003744.s009.tif]

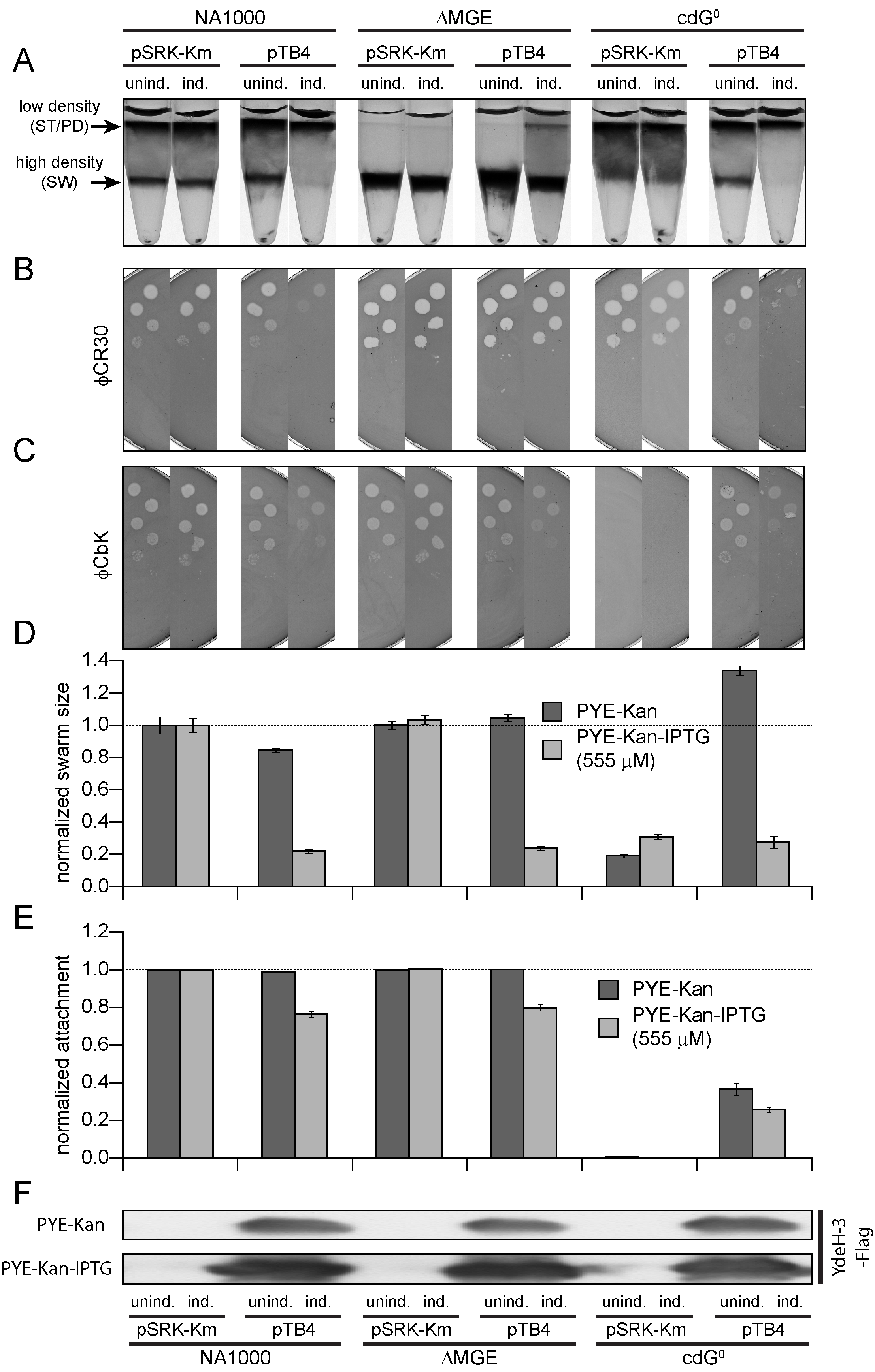

Supplement: Figure S10 — Cell density and φCR30 phage sensitivity are regulated by c-di-GMP via a mobile genetic element. Same as in Figure 6, but with all control strains. The wild type, the mobile genetic element mutant (ΔMGE) and the cdG0 strain are shown, either carrying a YdeH overexpression construct (pTB4) or the empty vector backbone (pSRK-Km), both either with full IPTG induction or without inducer. These were tested for the cell density switch (A), the φCR30 (B) and φCbK (C) sensitivity, motility (D), surface attachment (E) and YdeH-3×Flag expression (F). (TIF) [file pgen.1003744.s010.tif]

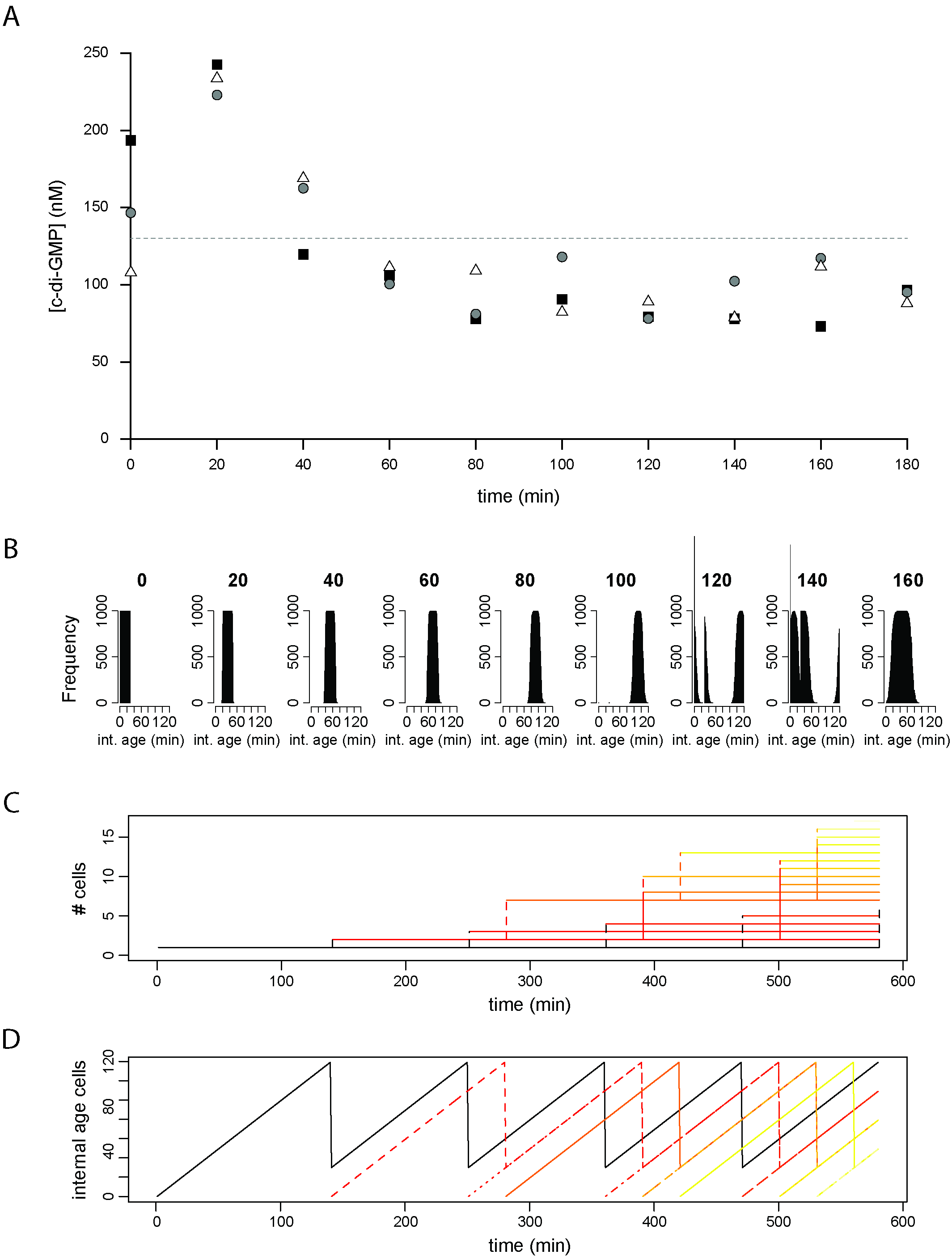

Supplement: Figure S11 — C-di-GMP fluctuations in synchronized C. crescentus populations. A) Quantification of the average intracellular c-di-GMP concentration of a synchronized C. cresentus population. Swarmer cells of three independent C. crescentus wild type cultures were harvested by density gradient centrifugation and followed for 180 min. Every 20 min the c-di-GMP concentration of the population is determined and the average intracellular c-di-GMP concentration is given in nM. The dotted line indicates the average c-di-GMP concentration in a mixed wild type population. B) Model of the internal age distribution of a synchronized C. crescentus population. Starting from a swarmer cell population, a snapshot of the internal age distribution is depicted every 20 min for a total of 160 min. C–D) Model of a C. crescentus population starting form a single cell. On the X-axis the total time of growth is given in minutes. On the Y-axis, either the number of cells (C) or the internal time in minutes (D) is given. The internal time starts at zero. When it reaches 137 minutes (tC) cells divide and the internal time is reset in both progeny to 0 minutes (swarmer cells) or ∼35 minutes (stalked cells, tS), respectively. Individual progeny are distinguishable by the color code and line structure. (TIF) [file pgen.1003744.s011.tif]
