# Supplementary material for: Bi-modal Distribution of the Second Messenger c-di-GMP Controls Cell Fate and Asymmetry during the Caulobacter Cell Cycle
Source: PLoS Genet. 2013 Sep 5;9(9):e1003744. doi: 10.1371/journal.pgen.1003744 (PMC3764195; doi:10.1371/journal.pgen.1003744)
Supplement: Table S1 — Strains, phages and plasmids used in this study. (PDF) [file pgen.1003744.s012.pdf]

Caulobacter crescentus strains:			
**Name**	**Genotype**	**Description**	**Source or Reference**
CB15	CB15	*C. crescentus* wild type ATCC 19089 Caulobacter vibrioides LOT: 3967454	(Poindexter and Cohen-Bazire, 1964)
NA1000	CB15N	Laboratory strain derived from CB15	(Evinger and Agabian, 1977)
SoA0071	CB15 Δ*popA*	markerless in frame deletion of *popA* in CB15 by allelic exchange using plasmid pAD8	this study
SoA0144	NA1000 Δ*cc0655*	markerless in frame deletion of *cc0655* in NA1000 by allelic exchange using plasmid pSA93	this study
SoA0145	NA1000 Δ*tipF*	markerless in frame deletion of *tipF* in NA1000 by allelic exchange using plasmid pSA94	this study
SoA0154	NA1000 Δ*cc0740*	markerless in frame deletion of *cc0740* in NA1000 by allelic exchange using plasmid pAD2	this study
SoA0155	NA1000 Δ*cc0857*	markerless in frame deletion of *cc0857* in NA1000 by allelic exchange using plasmid pSA91	this study
SoA0156	NA1000 Δ*cc0896*	markerless in frame deletion of *cc0896* in NA1000 by allelic exchange using plasmid pSA102	this study
SoA0157	NA1000 Δ*cc1086*	markerless in frame deletion of *cc1086* in NA1000 by allelic exchange using plasmid pSA79	this study
SoA0161	NA1000 Δ*cc3094*	markerless in frame deletion of *cc3094* in NA1000 by allelic exchange using plasmid pSA96	this study
SoA0162	NA1000 Δ*cc3148*	markerless in frame deletion of *cc3148* in NA1000 by allelic exchange using plasmid pSA90	this study
SoA0163	NA1000 Δ*dgcA*	markerless in frame deletion of *dgcA* in NA1000 by allelic exchange using plasmid pSA80	this study
SoA0166	CB15 Δ*cc0655*	markerless in frame deletion of *cc0655* in CB15 by allelic exchange using plasmid pSA93	this study
SoA0167	CB15 Δ*tipF*	markerless in frame deletion of *tipF* in CB15 by allelic exchange using plasmid pSA94	this study
SoA0168	CB15 Δ*cc0740*	markerless in frame deletion of *cc0740* in CB15 by allelic exchange using plasmid pAD2	this study
SoA0170	CB15 Δ*cc0896*	markerless in frame deletion of *cc896* in CB15 by allelic exchange using plasmid pSA102	this study
SoA0171	CB15 Δ*cc1086*	markerless in frame deletion of *cc1086* in CB15 by allelic exchange using plasmid pSA79	this study
SoA0173	CB15 Δ*dgcB*	markerless in frame deletion of *dgcB* in CB15 by allelic exchange using plasmid pAD7	(Abel et al., 2011)
SoA0175	CB15 Δ*cc3094*	markerless in frame deletion of *cc3094* in CB15 by allelic exchange using plasmid pSA96	this study

SoA0176	CB15 Δ*cc3148*	markerless in frame deletion of *cc3148* in CB15 by allelic exchange using plasmid pSA90	this study
SoA0177	CB15 Δ*dgcA*	markerless in frame deletion of *dgcA* in CB15 by allelic exchange using plasmid pSA80	this study
SoA0739	NA1000 pSA129	NA1000 containing pSA129	this study
SoA0741	CB15 pSA129	CB15 containing pSA129	this study
SoA0764	NA1000 Δ*dgcB* Δ*cc0857* Δ*cc0740* Δ*dgcA* Δ*pleD* Δ*cc0655* Δ*cc3094* Δ*cc0896* Δ*cc1086* Δ*cc0091* Δ*pdeA* Δ*cc3148*	markerless in frame deletion of *cc1086, cc0091, pdeA and cc3148* in NA1000 cdG^0 background by allelic exchange using plasmids pSA79, pSA156, pSA81, and pSA90, respectively; refered to as $rcdG^0$ strain in NA1000 background	this study
SoA0801	NA1000 pSRK-Km	NA1000 containing pSRK-Km	this study
SoA0803	CB15 pSRK-Km	CB15 containing pSRK-Km	this study
SoA0827	CB15 cdG^0 *dgcB* $^+$	the c-di-GMP free strain in CB15 background containing a chromosomal *dgcB* copy in the *dgcB* locus which was introcduce by general transduction using ϕCR30 and the marker CMS21 (West et al 2002).	this study
SoA0918	NA1000 Δ*cc0091*	markerless in frame deletion of *cc0091* in NA1000 by allelic exchange using plasmid pSA156	this study
SoA0930	CB15 Δ*cc0091*	markerless in frame deletion of *cc0091* in CB15 by allelic exchange using plasmid pSA156	this study
SoA1015	CB15 Δ*pleD* pSA164	CB15 Δ*pleD* containing pSA164	this study
SoA1061	CB15 Δ*pleD* pSA129	CB15 Δ*pleD* containing pSA129	this study
SoA1154	NA1000 cdG^0 pSA129	the c-di-GMP free strain in NA1000 background containing pSA129	this study
SoA1273	NA1000 Δ*dgcB* Δ*cc0857* Δ*cc0740* Δ*dgcA* Δ*cc0655* Δ*cc3094* Δ*cc0896* Δ*cc1086* Δ*cc0091* Δ*pdeA* Δ*cc3148*	$rcdG^0$ strain lacking the final markerless in frame deletion of *pleD* ; refered to as $rcdG^0$::*pleD*	this study
SoA1372	NA1000 cdG^0 pSRK-Km	the c-di-GMP free strain in NA1000 background containing pSRK-Km	this study
SoA1374	NA1000 cdG^0::*ydeH* pSRK-Km	the c-di-GMP free strain in NA1000 background containing a chromosomal copy of YdeH integrated by allelic exchange using plasmid pSA223 and pSRK-Km	this study
SoA1481	CB15 cdG^0 pTB4	the c-di-GMP free strain in CB15 background containing pTB4	this study
SoA1496	NA1000 cdG^0 pSA280	the c-di-GMP free strain in NA1000 background containing pSA280	this study
SoA1498	CB15 cdG^0 pSA280	the c-di-GMP free strain in CB15 background containing pSA280	this study
SoA1507	CB15 cdG^0 pSA129	the c-di-GMP free strain in CB15 background containing pSA129	this study
SoA1508	CB15 cdG^0 pSRK-Km	the c-di-GMP free strain in CB15 background containing pSRK-Km	this study

SoA1511	CB15 cdG^0::*ydeH* pSRK-Km	the c-di-GMP free strain in CB15 background containing a chromosomal copy of YdeH integrated by allelic exchange using plasmid pSA223 and pSRK-Km	this study
SoA1538	NA1000 ΔMGE	FC766; NA1000 with a deletion of the mobile genetic element between position 473069-499098 of the NA1000 genome	(Marks et al., 2002)
SoA1539	NA1000 ΔMGE pTB4	NA1000 ΔMGE containing pTB4	this study
SoA1541	NA1000 ΔMGE pSRK-Km	NA1000 ΔMGE containing pSRK-Km	this study
SoA1569	CB15 Δ*cc0857*	markerless in frame deletion of *cc0857* in CB15 by allelic exchange using plasmid pSA91	this study
SoA1587	rcdG^0::*ydeH*	chromoslomal integration of *ydeH* in rcdG^0 by alleic exchange using pSA223	this study
SoA1612	NA1000 *hfsA*$^+$ pSRK-Km	markerless exchange of *hfsA*$_{NA1000}$ to functional *hfsA* by allelic exchange using pSA178 in NA1000 containing pSRK-Km	this study
SoA1615	NA1000 *hfsA*$^+$ ΔMGE pSRK-Km	markerless exchange of *hfsA*$_{NA1000}$ to functional hfsA by allelic exchange using pSA178 in NA1000 ΔMGE containing pSRK-Km	this study
SoA1616	NA1000 cdG^0 *hfsA*$^+$ pSRK-Km	markerless exchange of *hfsA*$_{NA1000}$ to functional *hfsA* by allelic exchange using pSA178 in NA1000 cdG^0 containing pSRK-Km	this study
SoA1617	NA1000 *hfsA*$^+$ pTB4	markerless exchange of *hfsA*$_{NA1000}$ to functional *hfsA* by allelic exchange using pSA178 in NA1000 containing pTB4	this study
SoA1620	NA1000 *hfsA*$^+$ ΔMGE pTB4	markerless exchange of *hfsA* NA1000 to functional hfsA by allelic exchange using pSA178 in NA1000 ΔMGE containing pTB4	this study
SoA1621	NA1000 cdG^0 *hfsA*$^+$ pTB4	markerless exchange of *hfsA* NA1000 to functional hfsA by allelic exchange using pSA178 in NA1000 cdG0 containing pTB4	this study
SoA1624	NA1000 pSA266	NA1000 containing pSA266	this study
SoA1626	NA1000::CMS0	NA1000 containing a kanamycin resistance marker (CMS0) integrated via plasmid pLW132 linked to *cc0091*	(West et al., 2002)
SoA1627	rcdG^0::*ydeH* ::CMS0	marker CMS0 tranfered in rcdG^0::*ydeH* by general transduction using ϕCR30, the strain retained the *cc0091* deletion	this study
SoA1628	NA1000::*ydeH* ::CMS0 Δ*dgcB* Δ*cc0857* Δ*cc0740* Δ*dgcA* Δ*pleD* Δ*cc0655* Δ*cc3094* Δ*cc0896* Δ*cc1086* Δ*pdeA* Δ*cc3148*	marker CMS0 tranfered in rcdG^0::*ydeH* by general transduction using ϕCR30, the strain optained a wild-type *cc0091* allel	this study
SoA1632	NA1000::CMS12	NA1000 containing a kanamycin resistance marker (CMS12) integrated via plasmid pLW92 linked to *cc1086*	(West et al., 2002)
SoA1633	rcdG^0::*ydeH* ::CMS12	marker CMS12 tranfered in rcdG^0::*ydeH* by general transduction using ϕCR30, the strain retained the *cc1086* deletion	this study

SoA1634	NA1000::*ydeH* ::CMS12 Δ*dgcB* Δ*cc0857* Δ*cc0740* Δ*dgcA* Δ*pleD* Δ*cc0655* Δ*cc3094* Δ*cc0896* Δ*cc0091* Δ*pdeA* Δ*cc3148*	marker CMS12 tranfered in rcdG^0::*ydeH* by general transduction using ϕCR30, the strain optained a wild-type *cc1086* allel	this study
SoA1644	NA1000::CMS37	NA1000 containing a kanamycin resistance marker (CMS37) integrated via plasmid pLW124 linked to *pdeA*	(West et al., 2002)
SoA1645	rcdG^0::*ydeH* ::CMS37	marker CMS37 tranfered in rcdG^0::*ydeH* by general transduction using ϕCR30, the strain retained the *pdeA* deletion	this study
SoA1646	NA1000::*ydeH* ::CMS37 Δ*dgcB* Δ*cc0857* Δ*cc0740* Δ*dgcA* Δ*pleD* Δ*cc0655* Δ*cc3094* Δ*cc0896* Δ*cc1086* Δ*cc0091* Δ*cc3148*	marker CMS37 tranfered in rcdG^0::*ydeH* by general transduction using ϕCR30, the strain optained a wild-type *pdeA* allel	this study
SoA1648	rcdG^0 pBV-PA5295	rcdG^0 containing pBV-PA5295	this study
SoA1650	rcdG^0::ydeH pBV-PA5295	rcdG^0::ydeH containing pBV-PA5295	this study
SoA1652	rcdG^0::*pleD* pBV-PA5295	rcdG^0::*pleD* containing pBV-PA5295	this study
UJ2827	NA1000 Δ*popA*	markerless in frame deletion of *popA* in NA1000 by allelic exchange using plasmid pAD8	(Duerig et al., 2009)
UJ3252	NA1000 Δ*pilA*	deletion of *pilA* in NA1000	Assaf Levi
UJ3257	CB15 Δ*flgH*	markerless in frame deletion of *flgH* in CB15 by allelic exchange using plasmid pAL2	Assaf Levi
UJ4449	NA1000 Δ*dgcB*	markerless in frame deletion of *dgcB* in NA1000 by allelic exchange using plasmid pAD7	(Abel et al., 2012)
UJ4450	NA1000 Δ*pleD*	markerless in frame deletion of *pleD* in NA1000 by allelic exchange using plasmid pSA95	(Abel et al., 2012)
UJ4454	NA1000 Δ*pdeA*	markerless in frame deletion of *pdeA* in NA1000 by allelic exchange using plasmid pSA81	(Abel et al., 2012)
UJ4462	CB15 Δ*dgcB*	markerless in frame deletion of *dgcB* in CB15 by allelic exchange using plasmid pAD7	(Abel et al., 2012)
UJ4463	CB15 Δ*pleD*	markerless in frame deletion of *pleD* in CB15 by allelic exchange using plasmid pSA95	(Abel et al., 2012)
UJ4467	CB15 Δ*pdeA*	markerless in frame deletion of *pdeA* in CB15 by allelic exchange using plasmid pSA81	(Abel et al., 2012)
UJ4618	CB15 Δ*dgcB* Δ*cc0857*	markerless in frame deletion of *cc0857* in CB15 Δ*dgcB* by allelic exchange using plasmid pSA91	this study
UJ4760	CB15 Δ*dgcB* Δ*cc0857* Δ*cc0740*	markerless in frame deletion of *cc0740* in CB15 Δ*dgcB* Δ*cc0857* by allelic exchange using plasmid pAD2	this study
UJ4773	CB15 Δ*dgcB* Δ*cc0857* Δ*cc0740* Δ*dgcA*	markerless in frame deletion of *dgcA* in CB15 Δ*dgcB* Δ*cc0857* Δ*cc0740* by allelic exchange using plasmid pSA80	this study
UJ4774	CB15 pBV-MCS4	CB15 containing pBV-MCS4	this study
UJ4776	CB15 pBV-PA5295$_{E328A}$	CB15 containing pBV-PA5295$_{E328A}$	this study

UJ4778	CB15 pBV-PA5295	CB15 containing pBV-PA5295	this study
UJ4811	CB15 Δ*cc0740* Δ*cc0857* Δ*cc1850* Δ*cc3285* pAD5	CB15 Δ*cc0740* Δ*cc0857* Δ*cc1850* Δ*cc3285* containing pAD5	this study
UJ4816	CB15 Δ*dgcB* Δ*cc0857* Δ*cc0740* Δ*dgcA* Δ*pleD*	markerless in frame deletion of *pleD* in CB15 Δ*dgcB* Δ*cc0857* Δ*cc0740* Δ*dgcA* by allelic exchange using plasmid pSA95	this study
UJ4849	CB15 Δ*cc0740* Δ*cc0857* Δ*cc1850* Δ*cc2462* Δ*cc3285* pAD5	CB15 Δ*cc0740* Δ*cc0857* Δ*cc1850* Δ*cc2462* Δ*cc3285* containing pAD5	this study
UJ4929	CB15 Δ*dgcB* Δ*cc0857* Δ*cc0740* Δ*dgcA* Δ*pleD* Δ*cc0655*	markerless in frame deletion of *cc0655* in CB15 *ΔdgcB* Δ*cc0857* Δ*cc0740* Δ*dgcA* Δ*pleD* by allelic exchange using plasmid pSA93	this study
UJ4932	CB15 pAD5	CB15 containing pAD5	this study
UJ5061	CB15 ΔdgcB Δcc0857 Δcc0740 ΔdgcA ΔpleD Δcc0655 Δcc3094	markerless in frame deletion of *cc3094* in CB15 Δ*dgcB* Δ*cc0857* Δ*cc0740* Δ*dgcA* Δ*pleD* Δ*cc0655* by allelic exchange using plasmid pSA96	this study
UJ5065	NA1000 Δ*dgcB* Δ*cc0857* Δ*cc0740* Δ*dgcA* Δ*pleD* Δ*cc0655* Δ*cc3094* Δ*cc0896*	markerless in frame deletion of *pleD* in NA1000 Δ*dgcB* Δ*cc0857* Δ*cc0740* Δ*dgcA* Δ*cc0655* Δ*cc3094* Δ*cc0896* by allelic exchange using plasmid pSA95; refered to as cdG^0 strain in NA1000 background	this study
UJ5100	CB15 Δ*dgcB* Δ*cc0857* Δ*cc0740* Δ*dgcA* Δ*pleD* Δ*cc0655* Δ*cc3094* Δ*cc0896*	markerless in frame deletion of *cc0896* in CB15 Δ*dgcB* Δ*cc0857* Δ*cc0740* Δ*dgcA* Δ*pleD* Δ*cc0655* Δ*cc3094* by allelic exchange using plasmid pSA102; refered to as cdG^0 strain in CB15 background	this study
UJ5153	CB15 Δ*cc0655* Δ*cc0740* Δ*cc0857* Δ*cc0896* Δ*cc1850* Δ*cc2462* Δ*cc3094* Δ*cc3285* pAD5	CB15 Δ*cc0655* Δ*cc0740* Δ*cc0857* Δ*cc0896* Δ*cc1850* Δ*cc2462* Δ*cc3094* Δ*cc3285* containing pAD5	this study
UJ5154	CB15 Δ*cc0655* Δ*cc0740* Δ*cc0857* Δ*cc1850* Δ*cc2462* Δ*cc3285* pAD5	CB15 Δ*cc0655* Δ*cc0740* Δ*cc0857* Δ*cc1850* Δ*cc2462* Δ*cc3285* containing pAD5	this study
UJ5155	CB15 Δ*cc0655* Δ*cc0740* Δ*cc0857* Δ*cc1850* Δ*cc2462* Δ*cc3094* Δ*cc3285* pAD5	CB15 Δ*cc0655* Δ*cc0740* Δ*cc0857* Δ*cc1850* Δ*cc2462* Δ*cc3094* Δ*cc3285* containing pAD5	this study
UJ5170	NA1000 pTB4	NA1000 containing pTB4	this study
UJ5172	NA1000 cdG^0 pTB4	the c-di-GMP free strain in NA1000 background containing pTB4	this study
UJ5380	NA1000 pCM4	NA1000 containing pCM4	this study
UJ5381	NA1000 Δ*cc0655* Δ*cc0740* Δ*cc0857* Δ*cc0896* Δ*cc1850* Δ*cc2462* Δ*cc3094* Δ*cc3285* pCM4	NA1000 Δ*cc0655* Δ*cc0740* Δ*cc0857* Δ*cc0896* Δ*cc1850* Δ*cc2462* Δ*cc3094* Δ*cc3285* containing pCM4	this study
UJ5458	NA1000 pOP290-2	NA1000 containing pOP290-2	this study

UJ5459	NA1000 Δ*cc0655* Δ*cc0740* Δ*cc0857* Δ*cc0896* Δ*cc1850* Δ*cc2462* Δ*cc3094* Δ*cc3285* pOP290-2	NA1000 Δ*cc0655* Δ*cc0740* Δ*cc0857* Δ*cc0896* Δ*cc1850* Δ*cc2462* Δ*cc3094* Δ*cc3285* containing pOP290-2	this study
UJ5487	NA1000 pfljL-lacZ	NA1000 containing pfljL-lacZ	this study
UJ5488	NA1000 Δ*cc0655* Δ*cc0740* Δ*cc0857* Δ*cc0896* Δ*cc1850* Δ*cc2462* Δ*cc3094* Δ*cc3285* pfljL-lacZ	NA1000 Δ*cc0655* Δ*cc0740* Δ*cc0857* Δ*cc0896* Δ*cc1850* Δ*cc2462* Δ*cc3094* Δ*cc3285* containing pfljL-lacZ	this study
UJ7134	CB15 Δ*pleD* pSA217	CB15 Δ*pleD* containing pSA217	this study
UJ7137	CB15 cdG^0 pSA164	the c-di-GMP free strain in CB15 background containing pSA164	this study
UJ7138	CB15 cdG^0 pSA217	the c-di-GMP free strain in CB15 background containing pSA217	this study
UJ7156	CB15 $dgcB_{E261Q}$	CB15 with a mutation coding for the DgcB active site mutant $DgcB_{E261Q}$. Created by allelic exchange using plasmid pIH99	this study
UJ7162	CB15 cdG^0 $dgcB_{E261Q}$	the c-di-GMP free strain in CB15 background with a mutation coding for the DgcB active site mutant $DgcB_{E261Q}$. Created by allelic exchange using plasmid pIH99	this study

Escherichia coli strains:			
**Name**	**Genotype**	**Description**	**Source or Reference**
DH5α	(F-) F` *endA* 1 *hsdR* 17 (rK-mK plus) *glnV* 44 *thi* 1 *recA* 1 *gyr* Δ(Nal^R) *relA* 1 Δ(l*acIZYA* -*argF*)U169 *deoR* (Φ80dlac Δ(*lacZ*) M15)	Used for general cloning purposes	(Woodcock et al., 1989)
S17-1	RP4-2, Tc::Mu, KM-Tn7	Used as donor strain in conjugations to transfer oriT containing plasmids to *C. crescentus*	(Simon et al., 1983)

Phages:			
**Name**		**Description**	**Source or Reference**
ϕCbK		Bacteriophage that infects *C. crescentus* uses the pili as a receptor.	(Schmidt et al.,1965)
ϕCR30		Bacteriophage that infects *C. crescentus* uses the paracrystalline surface layer protein as a receptor; can be used for generalized transduction.	(Ely and Johnson, 1977)

Plasmids:			
**Name**		**Description**	**Source or Reference**
pAD2		pNPTS138; used for inframe deletion of *cc0740*	Anna Dürig
pAD5		pMR20; *popA -egfp* under control of *popA* promoter	(Duerig et al. 2009)
pAD7		pNPTS138; used for inframe deletion of *dgcB*	(Abel et al., 2011)
pAD8		pNPTS138; used for clean deletion of *popA*	(Duerig et al., 2009)
pAL2		pNPTS138; used for inframe deletion of *flgH*	Assaf Levi
pBV-MCS4		$Gent^R$, vanillate inducible medium-copy number plasmid	(Thanbichler et al., 2007)
pBV-PA5295		pBV-MCS4; *PA5292* under control of the vanillate promoter	(Duerig et al. 2009)
pBV-$PA5295_{E328A}$		pBV-MCS4; *PA5292* $_{E328A}$ active site mutant under the control of vanillate promoter	(Duerig et al. 2009)
pCM4		pRK-lacZ 290; *lacZ* under control of the *fliL* /*fliG* promoter	C. Mohr
pfljL-lacZ		pRK-lacZ 290; *lacZ* under control of the *fljL* promoter	E. Mangan
pIH99		pNPTS138; used for the introduction of *dgcB* $_{g781c}$ coding for $DgcB_{E261Q}$ by allelic exchange	this study
pLW124		Kan^R, suicide vector pBGS18T containing a homology region to *C. crescentus* for chromosomal intergration close to *pdeA*	(West et al., 2002)
pLW132		Kan^R, suicide vector pBGS18T containing a homology region to *C. crescentus* for chromosomal intergration close to *cc0091*	(West et al., 2002)
pLW92		Kan^R, suicide vector pBGS18T containing a homology region to *C. crescentus* for chromosomal intergration close to *cc1086*	(West et al., 2002)
pMR10		RK2 based Kan^R low copy number and broad host range vector with oriT	(Roberts et al., 1996)
pNPTS138		Kan^R, suicide vector with *sacB* gene and oriT	D. Alley
pOP290-2		pRK-lacZ 290; *lacZ* under control of the *fliF* promoter	K. Quon
pSA79		pNPTS138; used for inframe deletion of *cc1086*	this study
pSA80		pNPTS138; used for inframe deletion of *dgcA*	this study
pSA81		pNPTS138; used for inframe deletion of *pdeA*	(Abel et al., 2011)
pSA90		pNPTS138; used for inframe deletion of *cc3148*	this study
pSA91		pNPTS138; used for inframe deletion of *cc0857*	this study
pSA93		pNPTS138; used for inframe deletion of *cc0655*	this study
pSA94		pNPTS138; used for inframe deletion of *tipF*	this study
pSA95		pNPTS138; used for inframe deletion of *pleD*	(Abel et al., 2011)
pSA96		pNPTS138; used for inframe deletion of *cc3094*	this study

pSA102		pNPTS138; used for inframe deletion of *cc0896*	this study
pSA129		pMR10 lacking the lac Promoter	this study
pSA156		pNPTS138; used for inframe deletion of *cc0091*	this study
pSA164		pSA129; *pleD* under control of the *pleD* promoter	(Abel et al., 2011)
pSA178		pNPTS138; used for the introduction of a functional *hfA* allele by allelic exchange	this study
pSA217		pSA129; *pleD* $_{GG368DE}$ under control of the *pleD* promoter	this study
pSA223		pNPTS138; used for the introduction of lac promoter driven *ydeH* -3x*flag* by allelic exchange in the intergenic region between *cc3065* and *cc3066*	this study
pSA266		pSRK-Km; *ydeH* -*gfp* under control of the lac promoter	this study
pSA280		pSA129; *ydeH* -3x*flag* under control of the lac promoter	this study
pSRK-Km		Kan^R, IPTG inducible pBBR based medium-copy number plasmid	(Khan et al., 2008)
pTB4		pSRK-Km; *ydeH* -3x*flag* under control of the lac promoter	this study
