# Supplementary material for: Bi-modal Distribution of the Second Messenger c-di-GMP Controls Cell Fate and Asymmetry during the Caulobacter Cell Cycle
Source: PLoS Genet. 2013 Sep 5;9(9):e1003744. doi: 10.1371/journal.pgen.1003744 (PMC3764195; doi:10.1371/journal.pgen.1003744)
Supplement: Table S2 — Primers used for plasmid construction. (PDF) [file pgen.1003744.s013.pdf]

Primer name	Primer sequence	Restriction enzyme sits in 5' overhang	used for plasmid
1733	actagtcttctggacctcggccacgacg	SpeI	pSA79
1734	aagcttccagtaagcccgcaacccttg	HindIII	pSA79
1735	aagcttcgcgcgggcgcgtcagacag	HindIII	pSA79
1736	gaattcgctatgccgagggctgggcg	EcoRI	pSA79
1053	aaaactagtagccgtcagctccgacac	SpeI	pSA80
1054	aaaaagcttggccttacgggggaatcg	HindIII	pSA80
1055	aaaaagcttggccgccacccatggcgcccaacca	HindIII	pSA80
1056	aaagaattcccgatcaggtagaagccgtcc	EcoRI	pSA80
1069	aaaaagcttcggggccaggacatagtcgg	HindIII	pIH99
1072	aagaattctgcctgtcgagcgggcgaag	EcoRI	pIH99
1815	actagtaccgacgccaagatcaccag	SpeI	pSA90
1816	aagcttcgcaacgcccgcctccaaac	HindIII	pSA90
1817	aagctttccgcatgatcgtctcgtc	HindIII	pSA90
1818	gctagcacttcatgctgggcctgtcg	NheI	pSA90
1863	actagtgttctcgtccttgtccttgg	SpeI	pSA91
1864	aagcttctcgacacccttcatggtc	HindIII	pSA91
1865	aagcttgacgcgatcgacaccatc	HindIII	pSA91
1866	gaattcggcgagctggacctggtg	EcoRI	pSA91
1893	aagcttgcgttgatcttcagccactt	HindIII	pSA93
1894	ctgcagagccaaggcttgagtcttca	PstI	pSA93
1895	ctgcaggggttcctgaaggggtttg	PstI	pSA93
1896	gctagcatgatgatgcgatcaacgac	NheI	pSA93
1897	actagtaggtcttaacgtccgtcgaa	SpeI	pSA94
1898	aagcttcgtcccaagatcccttaagtc	HindIII	pSA94
1899	aagcttctggtaggacgcgactgct	HindIII	pSA94
1900	gaattcgcttgtttgaagcgggtct	EcoRI	pSA94
1905	actagtggcgtgtctgaggacaagat	SpeI	pSA96
1906	aagcttcgcagggtgaacattatacg	HindIII	pSA96
1907	aagcttgccctcctcacccgtagc	HindIII	pSA96
1908	gaattcctgaaaagcatcgccaagat	EcoRI	pSA96
1940	ggaattcgtggcaatgaaccgagagagtcaa	EcoRI	pSA102
1941	ccagacgtcaggacccgcggatccatccagcaccaccacgtacatcagcg	BamHI	pSA102
1942	ggatccgcgggtcctgacgtctgggatggggacttctgccgcgaggcc	BamHI	pSA102
1943	caagcttccagccaaggaccagagctatttc	HindIII	pSA102
2663	ctcgatcgaggtcccaatacgcaaaccgcctctccccgcgcgttggccgattcattaatgaagcttgtgtaggc tggagctgcttc	HindIII	pSA129
2664	aagcttggcgtaatcatggtcatagctgtttcctgtgtgaaattgttatccgctcacaatcatatgaatatcctcc ttag	HindIII	pSA129
1036	ggatccatgatctgtcgaacggccatc	BamHI	pSA156
1037	gaattccgttccttgagggtcactcgc	EcoRI	pSA156
1038	gaattcgagcaagaccagacgttccgc	EcoRI	pSA156
1039	gctagccggctgatcgtctgatccagt	NheI	pSA156
2958	gaattcgatttcgatcgccaagagcctcaa	EcoRI	pSA178
2959	cgaattcactagtgatagggtctgccagatcgggtt	EcoRI	pSA178
3369	aaaaactagtcaattggaattccgcaggaaaacatc	SpeI	pSA223

3370	ttttttcgaacaaaaaacccctcaagacccgtttagaggccccaaggggttatgctagctcttcgtccctgccttgt	BstBI	pSA223
3371	ttcgaaaaaacaaaaggctcagtcggaagactgggccttttgttttacaaggcagggacgaagag	BstBI	pSA223
3372	ttttcttaaggcgaccaagttcgtcaagat	AflII	pSA223
3589	aaaaggatcccgccgacgttgactattttt	BamHI	pSA227
3590	ttttggatccgcaagatttttccgtccgta	BamHI	pSA227
3591	aaaaaagctttgatgatctgctcgaccttg	HindIII	pSA228
3592	ttttaagcttctattcggcggcgatctt	HindIII	pSA228
3593	aaaaaagcttatgtctccgacgactggaag	HindIII	pSA229
3594	ttttaagcttagcatggggtcaaaaatctc	HindIII	pSA229
3595	aaaaaagcttgatgtggacggtttctttgg	HindIII	pSA230
3596	ttttaagcttgcgtacttctgctcgatgaa	HindIII	pSA230
3597	aaaaaagcttgccggtggagaacacttctt	HindIII	pSA231
3598	ttttaagcttccttgcagaagaacctctcg	HindIII	pSA231
926	cttgaagcttttacttgtacagctcgtccat	HindIII	pSA266
1323	catatggtgagcaagggcgag	NdeI	pSA266
4264	ataacaatttcacacaggaaacagcatatgatcaagaagacaacggaaat	NA	pSA266
4265	cccggtgaacagctcctcgcccttgctcacaactcggttaatcacattttgtt	NA	pSA266
2939	aaaagagctcataatgacacgatgcgaggaaacgcatatgatcaagaagacaacggaaat	SacI	pTB4
2940	aaaaggtaccagcagctccagcctacatta	KpnI	pTB4
5103	cgctacggcggccaggagttcgcgatgatcttcc	NA	pIH99
5104	ggaagatcatcgcgaactcctggccgccgtagcg	NA	pIH99
