# Supplementary material for: Bi-modal Distribution of the Second Messenger c-di-GMP Controls Cell Fate and Asymmetry during the Caulobacter Cell Cycle
Source: PLoS Genet. 2013 Sep 5;9(9):e1003744. doi: 10.1371/journal.pgen.1003744 (PMC3764195; doi:10.1371/journal.pgen.1003744)
Supplement: Text S1 — Construction of plasmids. (PDF) [file pgen.1003744.s014.pdf]

Bi-modal Distribution of the Second Messenger c-di-GMP Controls Cell Fate and Asymmetry During the *Caulobacter* Cell Cycle

Supporting Information Text S1

Sören Abel[1,2,#], Tabitha Bucher[1,3*], Micaël Nicollier[1,4*], Isabelle Hug[1], Volkhard Kaever[5], Pia Abel zur Wiesch[6], Urs Jenal[1,#]

[1] University of Basel, Biozentrum, 4054 Basel, Switzerland
[2] present address: Brigham and Women's Hospital/Harvard Medical School, Division of Infectious Diseases, 02115 Boston, MA, USA
[3] present address: Weizmann Institute of Science, Department of Molecular Genetics, 76100 Rehovot, Israel
[4] present address: Actelion Pharmaceuticals Ltd, Gewerbestrasse 16, 4123 Allschwil, Switzerland
[5] Hannover Medical School, Institute of Pharmacology, 30625 Hannover, Germany
[6] Brigham and Women's Hospital/Harvard Medical School, Global Health Equity, 02115 Boston, MA, USA
* Authors contributed equally to the manuscript
[#] For correspondence: sabel1@partners.org; urs.jenal@unibas.ch

Plasmid construction

Suicide plasmids for the generation of null alleles by double recombination pSA79, pSA80, pSA90, pSA91, pSA93, pSA94, pSA96, pSA102, and pSA156 were created by PCR amplification of a ~500-1000 bp long fragment up- and downstream of the region that was targeted for deletion from CB15 genomic DNA. Sequences of the used primers are given in table S2. The PCR fragments were cut with restriction enzymes as indicated in table S2 and all fragments were ligated at the same time into the likewise cut vector pNPTS138.

The allelic exchange vector pIH99 created by SOE-PCR amplification of two ~1000 bp long, partially overlapping fragment. The middle (overlapping) primers introduce the $dgcB_{g781c}$ mutation. The PCR products where mixed and amplified again using the non-overlapping primers from the first PCR round. The resulting ~2000 bp product was cut by EcoRI and HindIII (Table S2) followed by ligation into the likewise cut pNPTS138.

pSA217 was created by ligating the BamHI fragment of pSW8 [1] in likewise cut pSA164.

pSA178 was created by PCR amplification of the *hfsA* region with primer 2958 and 2959 from CB15 genomic DNA. The PCR product was cut with EcoRI and ligated into the likewise cut vector pNPTS138.

Chloramphenicol acetyl transferase (cat) was amplified by PCR using primer 2663 and 2664 from pKD3 and integrated into pMR10 by lambda red mediated recombination [2]. The resulting plasmid was cut by HindIII and the larger fragment was re-ligated to produce pSA129.

pTB4 was created by PCR amplification of YdeH with primer 2939 and 2940 from MG1655 genomic DNA. The PCR product was cut with SacI and KpnI and ligated into the likewise cut vector pSRK-Km.

For the creation of pSA223 two PCR fragments were amplified with primer 3369 and 3370 as well as 3371 and 3372 from CB15 genomic DNA. The PCR fragments were cut with restriction enzymes as indicated in table S2 and ligated with the ydeH containing BstBI fragment of pTB4 in AflII and SpeI cut pNPTS138.

pSA280 was created by ligating the YdeH containing T4-polymerase treated SalI, SacII fragment of pSA223 into EcoRV cut pSA129.

eGFP was amplified by PCR using primer 926 and 1323 from pSA115 [3]. The PCR product was cut with NdeI and HindIII and ligated into the likewise cut pSRK-Km. The resulting vector was opened again with NdeI and ydeH PCR amplified with primer 4264 and 4265 from pSA223 was integrated by isothermal assembly cloning [4].

The correct sequence of all plasmids was confirmed by sequencing.

1. Paul R, Weiser S, Amiot NC, Chan C, Schirmer T, et al. (2004) Cell cycle-dependent dynamic localization of a bacterial response regulator with a novel di-guanylate cyclase output domain. Genes Dev 18: 715–727. doi:10.1101/gad.289504.

2. Datsenko KA, Wanner BL (2000) One-step inactivation of chromosomal genes in Escherichia coli K-12 using PCR products. Proc Natl Acad Sci USA 97: 6640–6645. doi:10.1073/pnas.120163297.

3. Abel S, Chien P, Wassmann P, Schirmer T, Kaever V, et al. (2011) Regulatory cohesion of cell cycle and cell differentiation through interlinked phosphorylation and second messenger networks. Mol Cell 43: 550–560. doi:10.1016/j.molcel.2011.07.018.

4. Gibson DG, Young L, Chuang R-Y, Venter JC, Hutchison CA 3rd, et al. (2009) Enzymatic assembly of DNA molecules up to several hundred kilobases. Nat Methods 6: 343–345. doi:10.1038/nmeth.1318.
